# Prognostic and therapeutic roles of specific genotypes through target-gene sequencing on gastroenteropancreatic neuroendocrine carcinoma

**DOI:** 10.1093/oncolo/oyag185

**Published:** 2026-05-08

**Authors:** Jianwei Zhang, Panpan Zhang, Yanyan Zhang, Qiaomian Hu, Jie Li, Xiaotian Zhang, Jian Li, Xicheng Wang, Jun Zhou, Zhihao Lu, Zhi Peng, Yu Sun, Sha Wang, Ming Lu, Lin Shen

**Affiliations:** Department of Gastrointestinal Oncology, Key Laboratory of Carcinogenesis and Translational Research (Ministry of Education, Beijing, China), Peking University Cancer Hospital and Institute, Beijing 100143, China; Department of Radiation Therapy, National Cancer Center/National Clinical Research Center for Cancer/Cancer Hospital & Shenzhen Hospital, Chinese Academy of Medical Sciences and Peking Union Medical College, Shenzhen 518172, China; Department of Gastrointestinal Oncology, Key Laboratory of Carcinogenesis and Translational Research (Ministry of Education, Beijing, China), Peking University Cancer Hospital and Institute, Beijing 100143, China; Medical Department, Nanjing Geneseeq Technology Inc., Nanjing 210032, China; Medical Department, Nanjing Geneseeq Technology Inc., Nanjing 210032, China; Department of Gastrointestinal Oncology, Key Laboratory of Carcinogenesis and Translational Research (Ministry of Education, Beijing, China), Peking University Cancer Hospital and Institute, Beijing 100143, China; Department of Gastrointestinal Oncology, Key Laboratory of Carcinogenesis and Translational Research (Ministry of Education, Beijing, China), Peking University Cancer Hospital and Institute, Beijing 100143, China; Department of Gastrointestinal Oncology, Key Laboratory of Carcinogenesis and Translational Research (Ministry of Education, Beijing, China), Peking University Cancer Hospital and Institute, Beijing 100143, China; Department of Gastrointestinal Oncology, Key Laboratory of Carcinogenesis and Translational Research (Ministry of Education, Beijing, China), Peking University Cancer Hospital and Institute, Beijing 100143, China; Department of Gastrointestinal Oncology, Key Laboratory of Carcinogenesis and Translational Research (Ministry of Education, Beijing, China), Peking University Cancer Hospital and Institute, Beijing 100143, China; Department of Gastrointestinal Oncology, Key Laboratory of Carcinogenesis and Translational Research (Ministry of Education, Beijing, China), Peking University Cancer Hospital and Institute, Beijing 100143, China; Department of Gastrointestinal Oncology, Key Laboratory of Carcinogenesis and Translational Research (Ministry of Education, Beijing, China), Peking University Cancer Hospital and Institute, Beijing 100143, China; Department of Pathology, Key Laboratory of Carcinogenesis and Translational Research (Ministry of Education), Peking University Cancer Hospital and Institute, Beijing 100143, China; Medical Department, Nanjing Geneseeq Technology Inc., Nanjing 210032, China; Department of Gastrointestinal Oncology, Key Laboratory of Carcinogenesis and Translational Research (Ministry of Education, Beijing, China), Peking University Cancer Hospital and Institute, Beijing 100143, China; Department of Gastrointestinal Oncology, Key Laboratory of Carcinogenesis and Translational Research (Ministry of Education, Beijing, China), Peking University Cancer Hospital and Institute, Beijing 100143, China

**Keywords:** gastroenteropancreatic neuroendocrine carcinoma, genotype, next-generation sequencing, prognosis, therapeutics

## Abstract

**Purpose:**

Despite emerging genomic discoveries, few have been translated into practical management strategies for people with gastroenteropancreatic neuroendocrine carcinoma (GEPNEC). This study aims to identify key genes and their potential clinical relevance to improve care for this patient group.

**Methods:**

In the analytical cohort, we reported high-frequency alteration genes, significantly mutated genes, and driver genes and defined their overlap as key genes. Genotypes were identified as Type I/II/III. For further validation, we prospectively recruited eligible patients to build a main cohort, through which we performed univariate/multivariate Cox regression analysis and sketched Kaplan–Meier survival plot. Targetable genes were defined by the OncoKB database.

**Results:**

The analytical cohort consisted of 124 patients. The genes most frequently altered were TP53 (78%), RB1 (35%), APC (27%), and KRAS (17%). Key genes were identified as TP53, RB1, APC, and KRAS. The main cohort included 171 patients. We defined the genotype “Type I” (defined as TP53/RB1 co-alterations) as a reference. In midgut/hindgut NEC, we identified a “Type II” subtype characterized by mutations in either APC or KRAS alterations without TP53/RB1 co-alterations. Type II of midgut/hindgut NEC patients had elevated carcinoembryonic antigen (CEA) levels and longer median overall survival (mOS) than others (not reached vs 11.4 months, HR = 0.19[0.08, 0.44], *P* = .0028). In foregut NEC, we identified “Type III” (either APC/KRAS alterations or TP53/RB1 co-alterations). Type III of foregut NEC patients showed shorter mOS than others (11.6 vs 19.0 months, HR = 1.93[1.13, 3.25], *P* = .01). Type II patients who received non-platinum/etoposide (EP) chemotherapy had longer first-line progression-free survival (PFS) compared to EP regimen. They also benefited from second-line immunotherapy. Targeted therapy as second-line was also suitable for targetable patients (longer PFS than others: 12.5 vs 3.0 months, HR = 0.40[0.21-0.75], *P* = .0017).

**Conclusions:**

Our study identified key gene-based genotypes (Type II/III) of distinct GEPNEC patients and yielded their prognosis and therapeutic utility.

Implications for Practice These results advocate for the immediate integration of molecular genotyping into the standard diagnostic workup for GEPNEC. By stratifying patients into Type I, II, and III categories, clinicians can make evidence-based decisions regarding prognosis, first-line chemotherapy selection, optimal choice of immunotherapy and targeted agents. Our work ultimately aimed to improve both survival outcomes and quality of life for this heterogeneous patient population.

## Introduction

Poorly differentiated neuroendocrine carcinoma (NEC) is a highly aggressive tumor that can originate from almost any organ, with 2/3 of cases originating in the digestive system. The rapidly increasing incidence of NECs has led to a growing interest in understanding their heterogeneity in biological behavior, and confounding management has triggered increased attention. Patients with recurrent or metastatic GEPNEC have limited treatment options, with current first-line therapy generally following the management approach used in small cell lung cancer (SCLC).^1^^,^^2^ However, platinum/etoposide chemotherapy, commonly used for SCLC, is not as effective in GEPNEC. Second-line treatments also have limited efficacy, with a median overall survival of less than 6 months. Given the heterogeneity of GEPNECs, there is a need for specific treatment regimens tailored to patient groups.^1^

Studies on genomic alteration in the landscape of EP-NEC have rapidly gained importance.^3^^,^^4^ Next-generation target sequencing has revealed the comprehensive genomic landscapes of GEPNECs as a distinct entity, identifying driver genes and potential actionable aberrations.^5^ Unlike SCLC, TP53/RB1 co-inactivation is rare in GEPNECs. There is increasing evidence that GEPNECs have molecular features like adenocarcinomas. Professor Yachida et al. proposed a classification of pancreatic/non-pancreatic NECs into ductal-type and acinar-type based on multi-omics analyses.^6^^,^^7^ This raises the question of whether genetic features are associated with clinical information and the effectiveness of therapeutic strategies.^8–10^ Despite recent advances in understanding the molecular mechanisms of GEPNECs, genomic data that directly impact clinical management are still lacking.^11^

In this study, we investigated the clinical utility of genomic models. We identified TP53/RB1/KRAS/APC as key genes and established gene patterns to assess their prognostic and predictive roles. This research will pave the way for tailored therapies and precise management of GEPNECs.

## Methods

### Criteria of analytical cohort

Patients diagnosed as GEPNEC at Peking University Cancer Hospital from January 2010 to January 2020, with tumor DNA and ctDNA and adequate clinicopathological information, entered the “Analytical Cohort.” Baseline tumor specimens and plasma samples were collected. From these, key genes were defined by the intersection of high frequency and driver genes. Key gene-based genotype was explored through cox analysis of different patterns.

### Criteria of prospective cohort

We then prospectively recruited patients from January 2020 to January 2022 with key gene status to build the main cohort. It further explored the genotypes. Inclusion criteria for the main cohort: (1) Pathological diagnosis: It was scrupulously confirmed as NEC by 2 independent pathologists according to the 2010 World Health Organization Classification of Neuroendocrine Neoplasms and will be judged by the third pathologist when necessary.^12^^,^^13^ (2) Complete clinical information may be obtained from the Hospital Information System. (3) Aged 18 years or older. (4) Key gene status identified by NGS (tumor DNA). Exclusion criteria included: (1) unknown origins (for example: abdominal cavity). (2) pathological confirmed MiNEN. We retrieved informed consent from all patients in accordance with the ethical guidelines of the Helsinki Declaration. The study protocol was approved by the Institutional Review Boards of Peking University Cancer Hospital.

Due to the low incidence, we chose an external validation cohort from previous retrospective cohorts.^7^^,^^14^

### DNA extraction and sequencing

DNA was extracted from tissue, plasma, and lymphocytes using the QIAamp DNA Mini Kit, the QIAamp Circulating Nucleic Acid Kit (Qiagen) and the QIAamp DNA Blood Mini Kit (Qiagen), respectively. DNA concentration was evaluated with the Qubit fluorometer (Invitrogen, Carlsbad, CA, USA) and the Qubit dsDNA HS (High Sensitivity) Assay Kit. The ctDNA was evaluated by Agilent 2100 Bioanalyzer and the DNA HS kit (Agilent Technologies, Santa Clara, CA, USA).

DNA was fragmented, and libraries were prepared using Illumina TruSeq DNA Library Preparation Kit (Illumina, San Diego, CA) (tissue-DNA), and KAPA DNA Library Preparation Kit (Kapa Biosystems, Wilmington, MA, USA) (ctDNA). PCR pre-capture cycles were performed to generate sufficient fragments prior to hybridization. All libraries were hybridized to custom-designed biotinylated oligonucleotide probes (Roche NimbleGen, Madison, WI, USA) covering 425 cancer-associated genes (Table S12). DNA sequencing was carried out with the HiSeq Sequencing System (Illumina, San Diego, CA) with 2 × 151-bp paired-end reads.

### Sequencing analysis

After removing terminal adaptor sequences and low-quality data, the reads were mapped to the reference human genome by BWA (http://bio-bwa.sourceforge.net/) with default parameters. We used the NoahCare Toolkit using NCfilter (software developed by ourselves, version 1.5.0) for raw data quality control (QC), NCbamInfo (version 0.2.0) for QC alignment, NCanno (version 0.1.1) for annotation with multiple databases, and NChot (version 0.1.0) for hotspot region variant review. GATK (https://www.broadinstitute.org/gatk/, The Genome Analysis Toolkit) and MuTect were used to call for small insertions and deletions (indels) and single nucleotide variants (SNVs) in somatic DNA. Contra(http://contra-cnv.sourceforge.net) was used to detect copy number variants, and BreakDancer(https://omictools.com/breakdancer-tool) was used to detect cancer-associated structural variants.

### Key genes and targetable genes

The driver genes were calculated by unbiased driver genes dN/dS analysis (q ≤ 0.05).^15^ The threshold for high frequency mutations was above 10%. Significant variation genes were defined as differences between foregut and midgut/hindgut origins. The intersection of these genes yielded key genes. Targetable genes were determined based on therapeutic levels of OncoKB (1-3) (https://www.oncokb.org/, August 2022).

### Statistics

Tumor responses are assessed according to RECIST version 1.1. The objective response (OR) contains complete remission (CR) plus partial response (PR). Progression-free survival is the time from initiation of therapy to documented disease progression (PD) or death from any cause. The dichotomic variables of clinicopathologic and genomic characteristics were compared between the two groups using κ2 tests (or Fisher exact test when appropriate). Continuous variables between the two groups were compared using the t-test. We conducted the univariate/multivariate cox proportional hazard regression analysis and presented by Kaplan–Meier plot and log-rank tests. All statistical methods were performed with R software (version 4.1.4). Statistical significance was defined as *P* < .05, two-sided.

## Result

### Clinical and genetic features of GEPNEC

**Figure 1.**
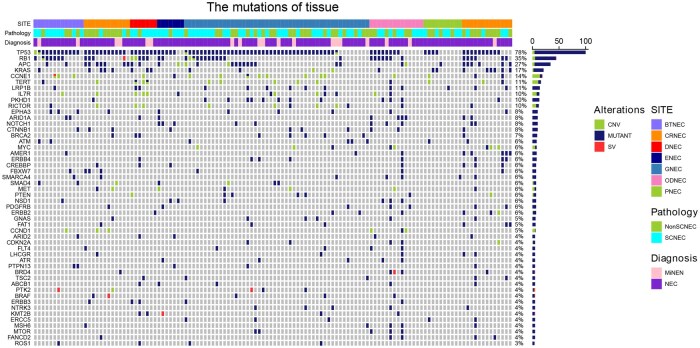
Oncoprint of comprehensive genomic landscape of GEPNEC. Genomic landscape of tumor DNA of the analytical cohort. Abbreviations: BTNEC=bile tract NEC, ENEC = Esophageal NEC, GNEC=gastric NEC, DNEC = duodenal NEC, CRNEC = colorectal NEC, ODNEC = Other digestive system-originated NECs (including liver, mesentery, and other suspected abdominal cavity origins), PNEC = pancreatic NEC. Traditional clinicopathological factors to predict prognosis were unsatisfactory. However, we observed a trend of longer OS in patients with APC mutation (see Table S4 and Figure S5).

The study flowchart was shown in Figure S1. The analytical cohort included 124 patients for genomic exploration. Of these, 62.9% of patients were male and 93.6% of patients were stage IV. Small cell types accounted for 54.0% of cases (Table S1). The oncoprint delineating the genomic landscape of tumor DNA and ctDNA was shown in Figure 1 and Figure S2.

In tumor DNA, we identified a total of 1108 variants across 96.0% (119/124) of patients. The top four altered genes were TP53 (78%), RB1 (35%), APC (27%), and KRAS (17%). These high-frequency alterations were also observed in ctDNA with relatively high concordance, as shown in Figure S3.

**Figure 2.**
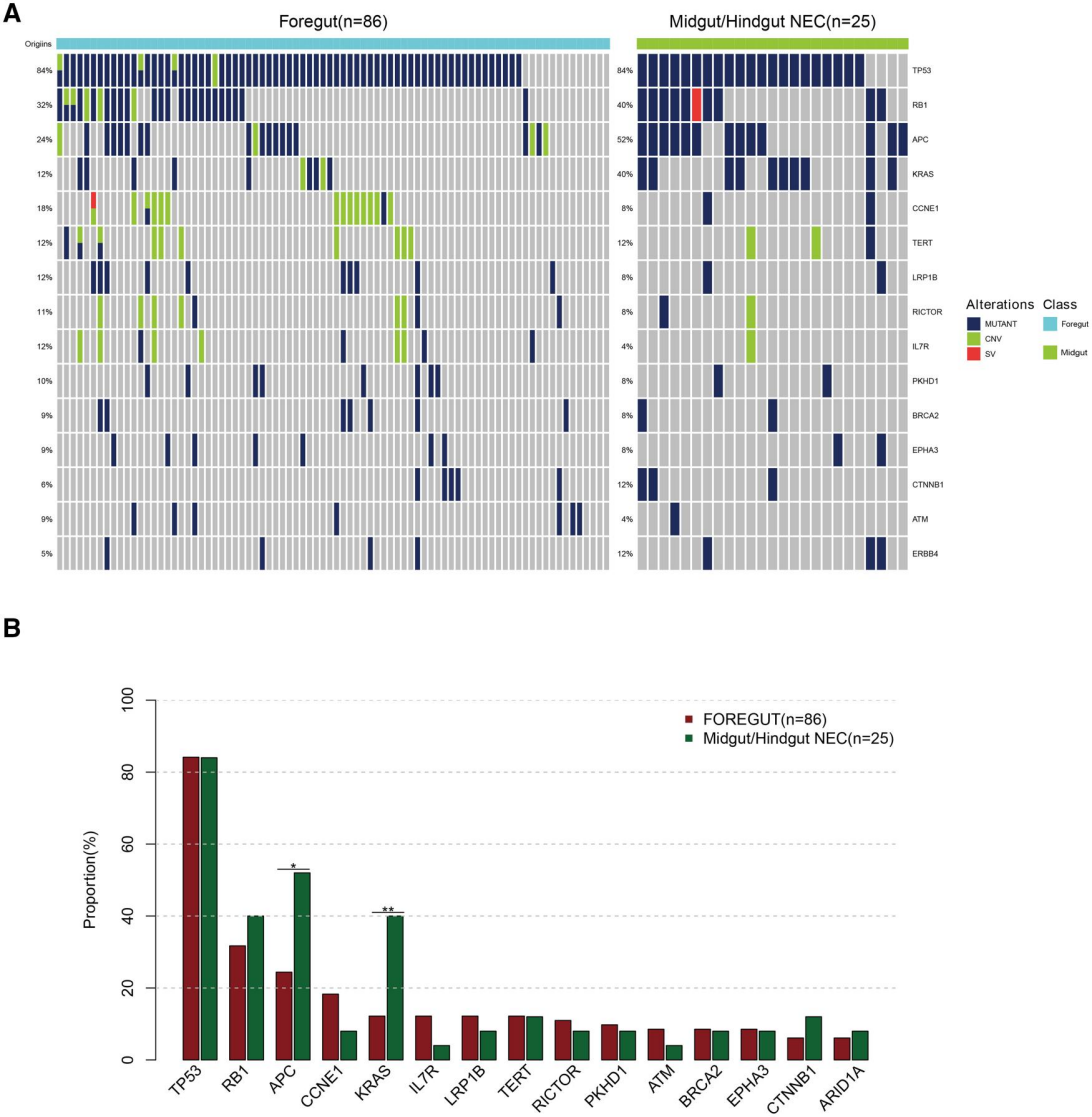
Genomic differences between foregut and midgut/hindgut NEC. (A) Genomic landscape disparity between foregut and midgut/hindgut NEC. (B) Top frequently mutated genes of between Foregut and midgut/hindgut NEC.

We calculated the driver genes and presented them in Table S3. Through Network-Venn analysis, TP53, RB1, APC, and KRAS were identified as frequently altered hub genes in various digestive adenocarcinomas and NEC^16^ (Table S2 and Figure S4). Foregut and midgut/hindgut NEC exhibit different clinical behaviors, and their genomic differences were compared. Higher frequency of KRAS and APC alterations in midgut/hindgut NEC compared to foregut NEC, while TP53 and RB1 were prevalent (see Figure S4 and Figure 2).

### Genotypes in GEPNEC

Based on our findings, we hypothesized that distinct genotypes may influence survival outcomes based on tumor origin. We conducted cox regression analysis to screen for all possible genotypes to identify the optimal ones, as presented in Table S5. We defined TP53/RB1 co-alteration as “Type I” gene pattern. In midgut/hindgut NEC (MH-NEC), we identified “Type II” pattern as either KRAS or APC alteration without TP53/RB1 co-alteration. In foregut NEC (F-NEC) patients, “Type III” pattern was defined as either KRAS/APC alteration or TP53/RB1 co-alterations.

The main cohort consisted of 171 GEPNEC patients, with 44 midgut/hindgut NEC and 127 foregut NEC patients. We observed that Type II patients were seen in 18/44 (40.9%) of midgut/hindgut NEC, who had elevated CEA levels and less metastatic burden. Type III patients accounted for 52/127 (40.9%), who had higher proportions of elevated NSE and systemic metastases (Table S6 and Table 1). Type I and Type III patients more likely had features of small-cell type, male, higher Ki67 and baseline NSE levels.

**Table 1.** Type II/Type III-based stratification of main cohort.

Level			Foregut NEC (*n* = 127)				Midgut/hindgut NEC (*n* = 44)				Main cohort (*n* = 171)
Gene pattern			Non-type III (*n* = 75)		Type III (*n* = 52)	*P*	Non-type II (*n* = 26)	Type II (*n* = 18)		*P*	
**Primary site (%)**		**Esophagus**	8 (10.7)	5 (9.6)		.107	–	–	-		13 (7.6)
		**Stomach**	39 (52.0)	21 (40.4)			–	–	-		60 (35.1)
		**Duodenum**	3 (4.0)	6 (11.5)			–	–	-		9 (5.3)
		**Pancreas**	16 (21.3)	8 (15.4)			–	–	-		24 (14.0)
		**Liver**	1 (1.3)	1 (1.9)			–	–	-		2 (1.2)
		**Bile tract**	8 (10.7)	11 (21.2)			–	–	-		19 (11.1)
		**Colon**	–	-		–	8 (30.8)	9 (50.0)	.33		17 (9.9)
		**Rectum**	–	-		–	18 (69.2)	9 (50.0)			27 (15.8)
**Age (mean [SD])**			60.53 (13.23)	61.40 (10.48)		.691	59.08 (11.19)	62.22 (8.10)	.313		60.75 (11.57)
**Pathology (%)**		**Non-SCNEC**	32 (42.7)	16 (30.8)		.16	18 (69.2)	13 (72.2)	1		79 (46.2)
		**SCNEC**	43 (57.3)	36 (69.2)			8 (30.8)	5 (27.8)			92 (53.8)
**Ki67 (mean (SD))**			0.75 (0.65,0.80)	0.80(0.70,0.90)		.31	0.80(0.70,0.83)	0.70(0.60,0.80)	.45		0.80(0.70,0.83)
**Gender (%)**		**Female**	19 (25.3)	23 (44.2)		.047	7 (26.9)	7 (38.9)	.611		56 (32.7)
		**Male**	56 (74.7)	29 (55.8)			19 (73.1)	11 (61.1)			115 (67.3)
**Stage (%)**		**I-III**	4 (5.3)	2 (3.8)		.753	1 (3.8)	1 (5.6)	.682		8 (4.7)
		**IV**	71 (94.7)	50 (96.2)			25 (96.2)	17 (94.4)			163 (95.3)
**Hepatic**		**No**	15 (20.0)	11 (21.2)		1	11 (42.3)	1 (5.6)	0.019		38 (22.2)
**metastases (%)**		**Yes**	60 (80.0)	41 (78.8)			15 (57.7)	17 (94.4)			133 (77.8)
**Lung**		**No**	68 (90.7)	45 (86.5)		.658	20 (76.9)	16 (88.9)	.539		149 (87.1)
**metastases (%)**		**Yes**	7 (9.3)	7 (13.5)			6 (23.1)	2 (11.1)			22 (12.9)
**Bone**		**No**	74 (98.7)	44 (84.6)		.007	24 (92.3)	16 (88.9)	1		158 (92.4)
**metastases (%)**		**Yes**	1 (1.3)	8 (15.4)			2 (7.7)	2 (11.1)			13 (7.6)
**NSE (%)^a^**		**No**	26 (45.6)	14 (30.4)		.171	10 (43.5)	7 (43.8)	1		57 (40.1)
		**Elevated**	31 (54.4)	32 (69.6)			13 (56.5)	9 (56.2)			85 (59.9)
**CA199 (%)^a^**		**No**	44 (81.5)	32 (72.7)		.43	19 (79.2)	11 (85.7)	.945		106 (77.4)
		**Elevated**	10 (18.5)	12 (27.3)			5 (20.8)	3 (14.3)			31 (22.6)
**CEA (%)^a^**		**No**	42 (77.8)	33 (71.7)		.643	19 (79.2)	8 (50.0)	.052		101 (72.1)
		**Elevated**	12 (22.2)	13 (28.3)			5 (20.8)	8 (50.0)			39 (27.9)
**Surgery**		**No**	40 (54.1)	29 (55.8)		.993	9 (37.5)	8 (44.4)	.892		89 (52.0)
**history (%)**		**Yes**	34 (45.9)	23 (44.2)			15 (62.5)	10 (55.6)			82 (48.0)
**First-line (%)^b^**		**-**	3 (4.0)	3 (5.8)		.731	1 (3.8)	1 (5.5)	.294		8 (4.7)
		**EP**	49 (65.3)	37 (71.1)			13 (50.0)	12 (66.7)			111 (68.1)
		**IP**	8 (10.7)	4 (7.7)			5 (19.2)	2 (11.1)			19 (11.7)
		**CAPTEM**	2 (2.7)	0 (0.0)			0 (0.0)	1 (5.5)			3 (1.8)
		**Other Chemotherapy**	12 (16.0)	7 (13.5)			7 (26.9)	2 (11.1)			28 (17.2)
		**Other treatment**	1 (1.3)	1 (1.9)			0 (0.0)	0 (0.0)			2 (1.2)
**Second-line (%)^c^**		**EP**	4 (7.5)	4 (11.8)		.954	1 (5.6)	0 (0.0)	.48		9 (7.6)
		**IP**	4 (7.5)	3 (8.8)			3 (16.7)	1 (7.1)			11 (9.2)
		**CAPTEM**	1 (1.9)	1 (2.9)			0 (0.0)	0 (0.0)			2 (1.7)
		**Other Chemotherapy**	22 (41.5)	13 (38.2)			6 (33.3)	9 (64.3)			50 (42.0)
		**Immunotherapy**	18 (34.0)	12 (35.3)			6 (33.3)	3 (21.4)			39 (32.8)
		**Target therapy**	1 (1.9)	0 (0.0)			2 (11.1)	1 (7.1)			4 (3.4)
		**Other treatment**	3 (5.7)	1 (2.9)			0 (0.0)	0 (0.0)			4 (3.4)
**First-line efficacy**		**Response**	41 (62.1)	31 (66.0)		.826	15 (62.5)	14 (82.4)	.304		101 (65.6)
**(%)^d^**		**Non-response**	25 (37.9)	16 (34.0)			9 (37.5)	3 (17.6)			53 (34.4)
**Second-line**		**Response**	34 (72.3)	26 (81.2)		.521	10 (62.5)	9 (81.8)	.515		79 (73.8)
**efficacy (%)^e^**		**Non-response**	13 (27.7)	6 (18.8)			6 (37.5)	2 (18.2)			27 (26.2)

a 26 patients had no baseline NSE level.

b Only adjuvant chemotherapy.

c There were 40 patients in F-NEC with no second-line information and 12 patients had no second-line information in MH-NEC.

d There were 14 patients in F-NEC had no confirmed evaluation, and the number was 3 in MH-NEC.

e There were 48 patients in F-NEC had no confirmed evaluation, and the number was 17 in MH-NEC.

### Prognostic roles of genotypes

**Figure 3.**
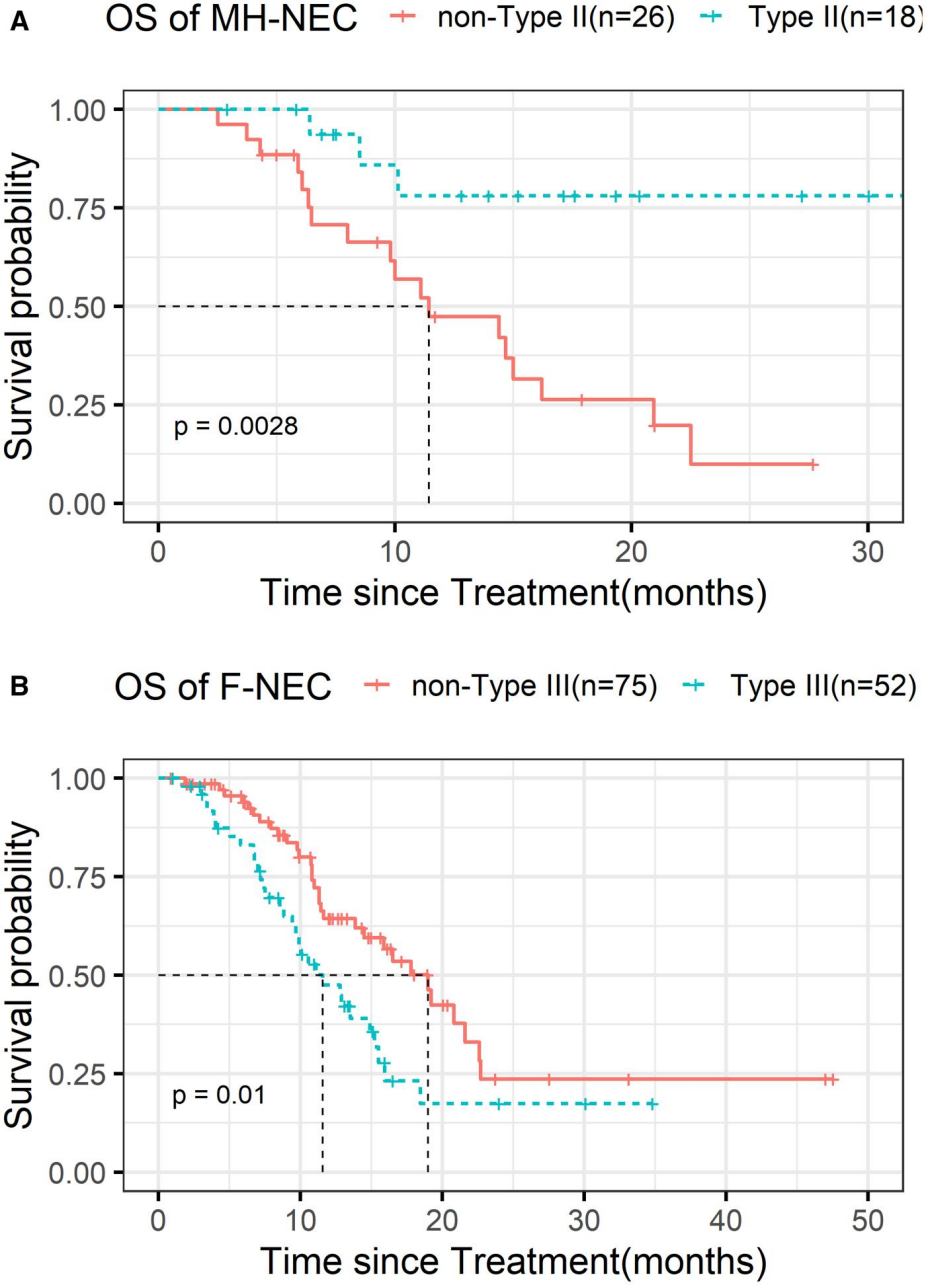
Kaplan–Meier survival curves of overall survival stratified by Type II/Type III in midgut/hindgut-NEC and foregut-NEC. Kaplan–Meier plot of overall survival of Type II on midgut/hindgut-NEC patients (A), and of Type III on foregut-NEC (B). Abbreviations: MH-NEC: Midgut/hindgut NEC; F-NEC: Foregut NEC.

Type II patients showed significantly longer median overall survival (mOS) than non-Type II patients (not reached vs 11.4 months, HR = 0.19(0.08, 0.44), *P* = .0028). The longer OS observed in Type II patients resembled that of colorectal adenocarcinoma, which prompted further investigation. For Type III patients, mOS was 11.6 months compared to 19.0 months for non-Type III patients, with a HR of 1.93(1.13, 3.25) and a *P*-value of .01 (Table 2). In comparison, the association between Type I and prognosis was not that significant (Figure S7). In the external validation cohort:^7^^,^^14^ Type III foregut NEC was also associated with poorer survival (Type III vs. non-Type III: 13.0 vs. 28.0 months, *P* = .0041) (Figure 3 and Figure S6).

**Table 2.** Univariate/multivariate Cox regression analysis of prognosis of main cohort.

		F-NEC			MH-NEC		
Stratification	Factors	Hazard ratio	CI95	*P*-value	Hazard ratio	CI95	*P*-value
**Univariate analysis**							
**Site**	Bile duct vs Esophagus	0.47	0.17-1.32	.153	–	–	–
	Stomach vs Esophagus	0.61	0.27-1.35	.22	–	–	–
	Duodenum vs Esophagus	1.75	0.6-5.13	.305	–	–	–
	Pancreas vs Esophagus	0.7	0.29-1.68	.426	–	–	–
	Liver vs Esophagus	1.43	0.3-6.79	.655	–	–	–
	Rectum vs Colon	–	–	–	1.11	0.45-2.77	.817
**Gender**	Male vs Female	1.01	0.99-1.03	.429	1.01	0.97-1.05	.502
**Age**		2.35	0.37-15	.368	12.86	0.43-381.74	.14
**Pathology**	SCNEC vs non-SCNEC	1.57	0.94-2.63	.087	0.98	0.39-2.44	.957
**Diagnosis**	NEC vs MiNEN	1.06	0.62-1.82	.823	0.97	0.37-2.51	.95
**Ki67**		Inf	0-Inf	.996	1.18	0.15-9.04	.874
**Ki67**	>55% vs <55%	1.72	0.81-3.63	.155	1.96	0.65-5.88	.232
**Hepatic metastases**	Yes vs No	2.14	1.01-4.51	.046	0.62	0.26-1.5	.291
**Lung metastases**	Yes vs No	1.46	0.66-3.22	.349	1.57	0.56-4.42	.389
**Bone metastases**	Yes vs No	3.09	1.38-6.91	.006	0.47	0.1-2.17	.336
**Elevated NSE**	Yes vs No	1.91	1.06-3.44	.03	2.09	0.8-5.46	.131
**Elevated CA199**	Yes vs No	2.05	1.14-3.71	.017	1.37	0.45-4.14	.58
**Elevated CEA**	Yes vs No	1.38	0.77-2.46	.279	0.88	0.32-2.47	.815
**TMB**	Low vs High	0.6	0.36-1.18	.104	0.88	0.37-2.11	.416
**Surgery history**	Yes vs No	0.68	0.38-1.22	.193	0.52	0.11-2.37	.441
**First-line**	CAPTEM vs EP	0.96	0.45-2.05	.91	0.62	0.17-2.26	–
	Chemo vs EP	0.42	0.06-3.1	.397	2.45	0.31-19.49	.516
	Other regimens vs EP	0.79	0.4-1.58	.503	1.03	0.33-3.21	–
**Second-line^a^**	ICI/Target vs Chemo	0.99	0.56-1.77	.978	1.76	0.56-5.49	.881
**Type I**	Yes vs No	1.59	0.95-2.67	.077	2.91	1.22-6.95	.037
**Type II**	Yes vs No	1.98	0.92-4.24	.08	0.19	0.06-0.64	.024
**Type III**	Yes vs No	1.95	1.18-3.23	.009	0.69	0.27-1.78	–
**Multivariate analysis**							
**Type I**	Yes vs No	1.38	0.74-2.58	.310	1.38	0.51-3.69	.524
**Type II**	Yes vs No	–	–	–	0.22	0.05-0.88	.033
**Type III**	Yes vs No	1.90	1.07-3.36	.028	–	–	–
**Pathology**	SCNEC vs Non-SCNEC	1.18	0.65-2.14	.588	0.74-	0.29-1.91	.539
**Hepatic metastases**	Yes vs No	2.29	0.99-5.29	.052	–	–	–
**Bone metastases**	Yes vs No	2.00	0.78-5.11	.146	–	–	–
**Elevated CA199**	Yes vs No	1.62	0.83-3.16	.159	–	–	–

Abbreviations: ICI: immunotherapy. Target: target therapy. Chemo: chemotherapy. CI: confidential interval. Non-SCNEC, Non-small cell neuroendocrine carcinoma; SCNEC, small cell neuroendocrinal carcinoma.

a Since the collinearity of Type I and Type III, we did not have the chance to put these in the multivariate-Cox analysis together. So, the 0.006 referred to the *P*-value when surgery and Type I was calculated.

**Figure 4.**
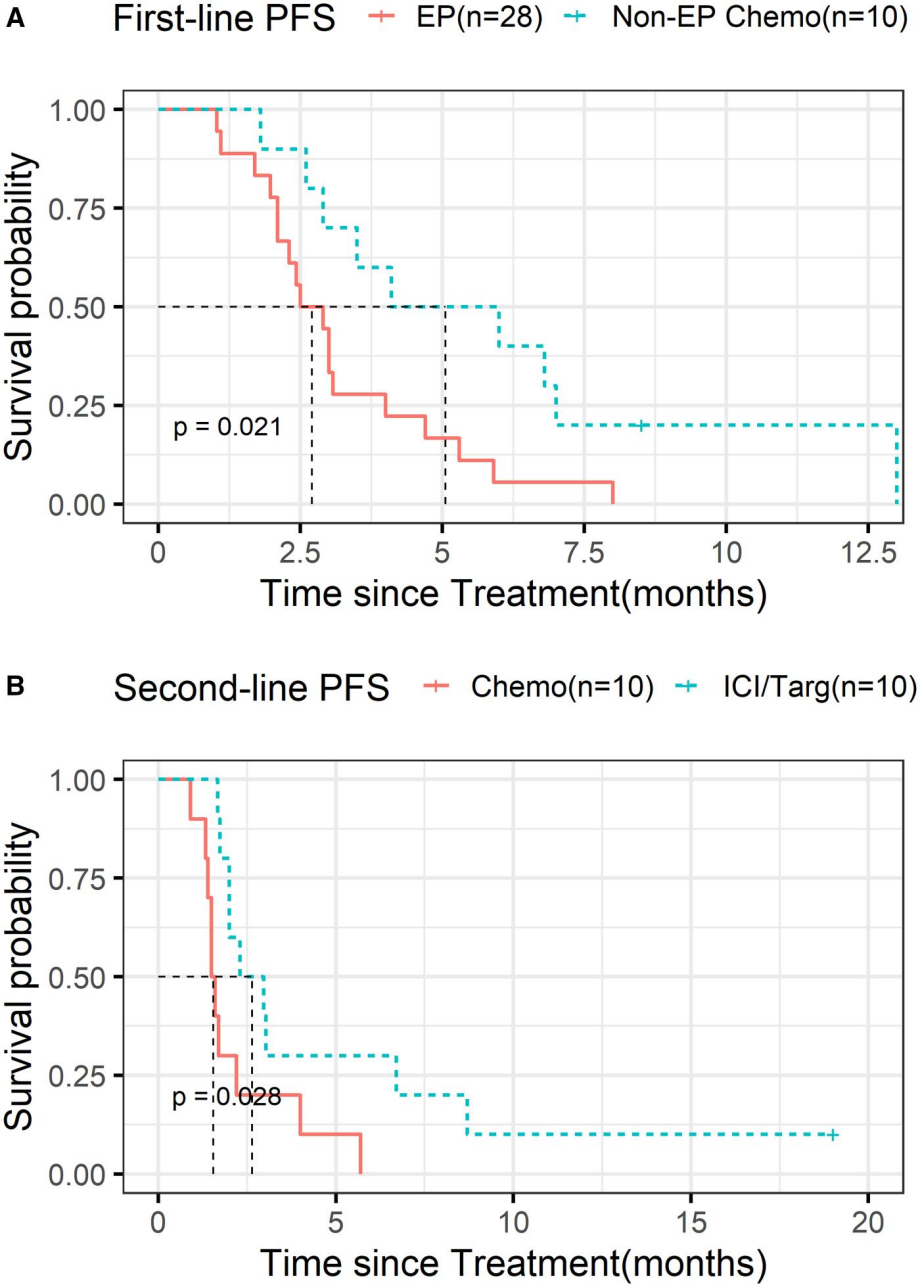
Kaplan–Meier survival curves of PFS of Type II patients receiving different first-line/second-line therapy. First-line PFS of EP vs non-EP chemotherapy (A) and second-line PFS of chemotherapy vs immunotherapy/target therapy (B)*. Abbreviation: chemo=chemotherapy, EP = etoposide/platinum. ICI/Targ = immunotherapy/target therapy.

Next, we investigated the therapeutic utility of these genotypes (see Table S11). Type II GEPNEC patients (*n* = 30) receiving first-line non-EP chemotherapy (irinotecan or oxaliplatin-based regimens) had significantly longer PFS compared to those receiving standard platinum/etoposide therapy (EP vs non-EP chemotherapy: 5.05 vs 2.70 months, HR = 0.42(0.20-0.90), *P* = .021) (Figure 4 and Table S7).

Since there is currently no confirmed recommendation for second-line therapy, we explored 2 potential treatments: chemotherapy, mainstay and immunotherapy/target therapy, the new trend. Interestingly, Type II patients achieved significantly longer PFS with immunotherapy/target therapy compared to chemotherapy (2.64 vs 1.55 months, HR = 0.40(0.14,0.99), *P* = .028, Figure 4 and Table S8).

**Figure 5.**
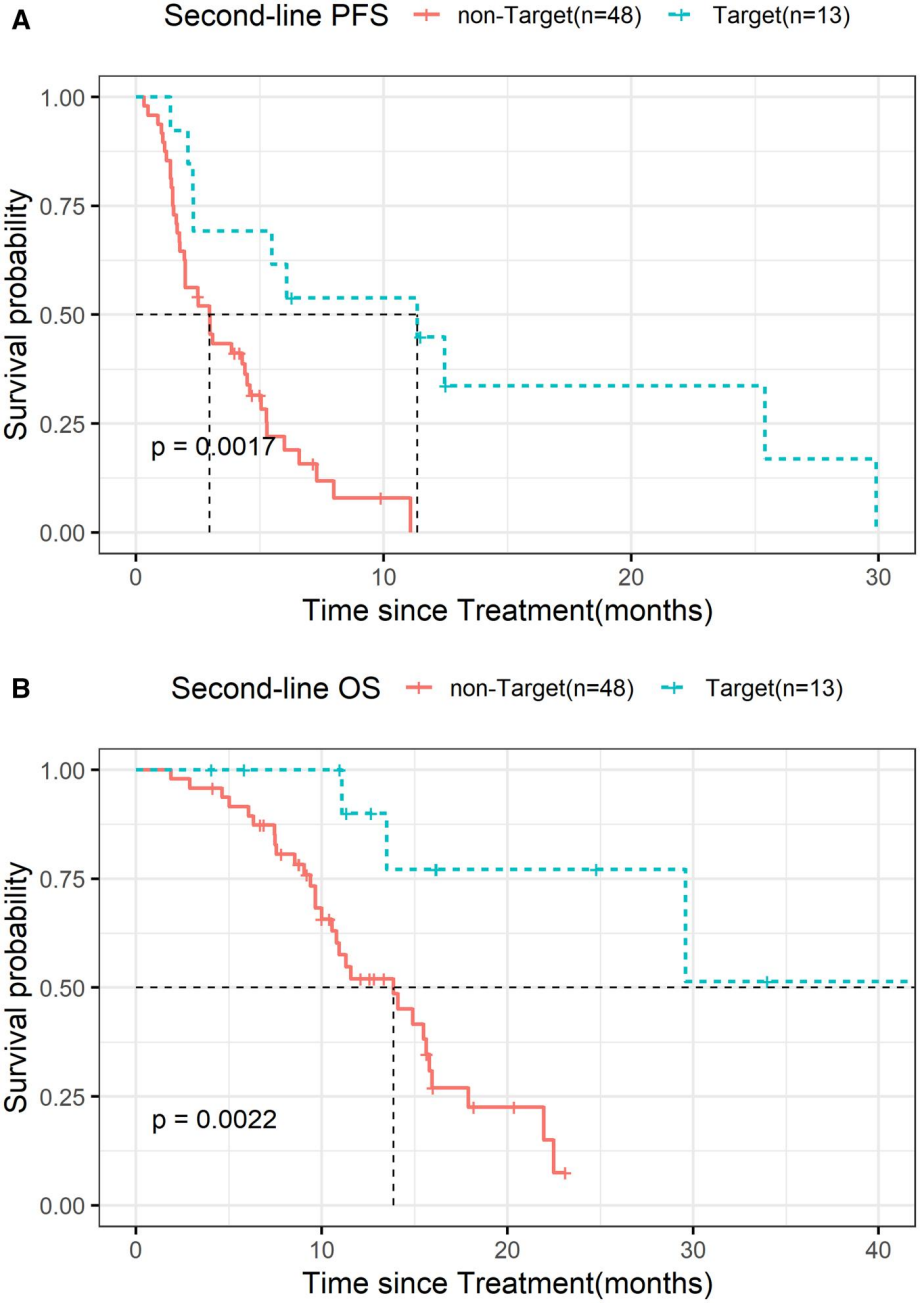
Kaplan–Meier survival curves demonstrating targeted therapy with promising survival benefit in second-line treatment. Kaplan–Meier plot of second-line PFS (A) and OS (B) of patients with target therapy and non-target therapy. Annotation: non-Target: Patients without targetable genes receiving regimens other than target therapy. Target: Patients with targetable genes receiving target therapy.

Whether genes guide target therapy in GEPNEC has attracted more attention. Approximately 44.4% of patients had druggable genes (Table S9), and the proportions rose to 55.6% (*n* = 69) when microsatellite instability (MSI, 2.9%) and tumor mutation burden-high (TMB-H, > 9.9muts/Mb, 17.1%). Targetable patients receiving target therapy achieved higher ORR, longer PFS and longer OS (target therapy with non-target therapy: ORR: 42.9% vs 25.0%, *P* = .33; PFS: 12.5 vs 3.0 months, HR = 0.40(0.21-0.75), *P* = .0017; OS: 42.9 vs 13.9 months, HR = 0.28(0.12-0.65), *P* = .0022). (Figure 5 and Table S10).

## Discussion

Compared to SCLC, clinical features of GEPNEC exhibit significant variability, leading to a debate about whether it should be managed similarly to SCLC. SCLC has a distinct driver gene pattern of TP53/RB1 co-alteration with clinical implications.^17^ Our study concentrates on the correlation between clinical characteristics and genetic profiles of GEPNEC.

Previous studies have primarily focused on the genomic landscape of GEPNEC, but the implications of key genes remained uncertain. The identified genes must meet certain criteria: high frequency, acting as drivers, and significance across subtypes. Low-frequency genes may weaken the statistical power of analysis, and only key genes can truly represent characteristics. Such distinctions can be found between midgut and pancreatic NET.^5^^,^^11^

We found Type I and Type III exhibit classic neuroendocrine-like features with a poorer prognosis. TP53/RB1 co-alteration pattern is a critical driver for subclassification of all lung NEC.^18–20^ RB1 bi-allelic inactivation and CNV had been reported as prognostic factors indicating worse OS for gastrointestinal NEC, yet the sample size was relatively small.^21^^,^^22^ Type III added KRAS/APC alterations to those seen in Type I, but better described poor prognosis. Non-type III patients had other frequent alteration genes such as CCNE1/MYC amplification (35.6%). CCNE1 amplified primarily in gastric NEC (64.7%).^7^ Functional TP53 and RB1 loss along with MYC alteration and active Akt status may generate lung and prostate NEC.^21^

Clinical features of Type II resembled colorectal adenocarcinomas,^8^ as indicated by long OS. A previous study suggested that CRNEC shared common top genes with colorectal adenocarcinomas: KRAS and APC mutations instead of TP53/RB1.^9^ midgut/hindgut NEC showed different mechanisms in preclinical models. Colonic epithelium-deriving LGR5+ organoids with TP53/RB1 knock-out did not induce morphological transition or neuroendocrine differentiation, requiring distinct drivers to enact a NEC fate.^23^ APC is a tumor‐suppressor gene that acts as a gatekeeper of the Wnt/β‐catenin pathway and initiates adenomatous polyposis and adenocarcinoma carcinogenesis.^4^ KRAS is an oncogene of the MAPK (mitogen-activated protein kinase) pathway, contributing to tumor cell growth and new blood vessel formation.^24^ APC and KRAS mutations are synergistic in intestinal tumor formation and progression.^25^ Several lung LCNEC studies have identified the “adenocarcinoma-like” subtype as the presence of KRAS/STK11/KEAP1 mutations with no TP53/RB1 co-alterations.^26^^,^^27^

For first-line regimens, chemotherapy outperformed standard EP in Type II GEPNEC, suggesting a preference for adenocarcinoma chemotherapy in such populations. We advocated clinical trials to optimize the management of Type II patients. Previous explorations have concentrated on whether SCNEC predicted a better response to EP. But it posed challenges for pathologists with 20%-30% of misdiagnosis of SCNEC and LCNEC.^28^ Pathological Rb loss and KRAS mutation were associated with response to platinum-based chemotherapy in pancreatic NEC.^29^^,^^30^ Our study revealed the complexity and integrated the benefits of both genes to produce better predictive effects. The CIRCAN-NEC study identified 10 GEPNEC patients harboring KRAS, APC, BRAF, or SMAD4 mutations as “adenocarcinoma-like subtypes.” They showed a poorer response to first-line EP.^8^ Meanwhile, patients with a RB1 mutation had worse PFS when they received the FOLFIRI regimen. Further results can be expected from the FOLFIRINEC trial, which will compare the efficacy of mFOLFIRINOX and EP regimen as first-line treatment in GEPNEC patients with different genomic characteristics^31^ (ClinicalTrials: NCT04325425). A retrospective study suggested that choosing regimens according to genotype was in lung LCNEC.^19^ The determination of second-line therapies is an unmet medical need in GEPNEC. Although EP rechallenge was reported to be the most effective second-line treatment for those with initial long response,^32^ other evidence was limited. Type II tended to benefit from second-line immunotherapy regimens, pending large-sample studies to explore the mechanism.

Druggable genes were found in large proportions, which was consistent with previous studies (49%-72.7%).^5^^,^^10^^,^^22^ The application of targetable genes should not be underestimated, not limited to the BRAF V600E mutation, MEK amplification, Wee1, and Aurora A kinase, which cut a striking figure in extra-pulmonary NEC.^33–36^ Notably, we found that the DDR pathway (BRCA1/2, ATM, ATR, PALB2, etc.) was relatively highly mutated in GEPNEC. Poly adenosine diphosphate–ribose polymerase (PARP) inhibitors target the pathway, yet they were not given sufficient attention. Taken together, precise medical evidence was provided for GEPNEC. Biomarkers for predicting the response to immunotherapy-treating GEPNEC were still in difficulty, as NEN is characterized by the lowest TMB of all tumors.^37^ The association between KRAS/APC alteration and immunotherapy was unknown,^38^ while TP53/RB1 co-alteration only indicated good efficacy for bladder urothelial carcinomas.^39^^,^^40^ ARID1A, a chromatin modification-associated gene, can predict longer PFS.^41^^,^^42^

We inevitably have some limitations. First, this is a single-center study, although the high-volume institution with large samples convinced the study. Subpopulations for analysis were limited by the low incidence of the disease. We did not have enough samples to set both the training/validation set. But we did some external validation.

## Conclusion

In summary, we generated key gene-based genotypes. Type II midgut/hindgut NEC resembled colorectal adenocarcinoma with a better prognosis. Type III foregut NEC had a poorer prognosis. Non-EP chemotherapy as first-line treatment and immunotherapy as second-line therapy were also effective in Type II. Second-line target therapy can be applied to targetable patients.

## Supplementary Material

oyag185_Supplementary_Data

## Data Availability

The data that support the findings of this study are available on request from the corresponding author upon reasonable request.

## References

[1] Frizziero M , Kilgour E, Simpson KL, et al Expanding therapeutic opportunities for extrapulmonary Neuroendocrine Carcinoma. *Clin Cancer Res*. 2022;28:1999-2019. 10.1158/1078-0432.CCR-21-3058 35091446 35091446 PMC7612728

[2] Fazio N , Spada F, Giovannini M. Chemotherapy in gastroenteropancreatic (GEP) neuroendocrine carcinomas (NEC): a critical view. Cancer Treat Rev. 2013;39:270-274. 10.1016/j.ctrv.2012.06.009 22819619

[3] Dasari A , Shen C, Halperin D, et al Trends in the incidence, prevalence, and survival outcomes in patients with neuroendocrine tumors in the United States. JAMA Oncol. 2017;3:1335-13420. 10.1001/jamaoncol.2017.0589 28448665 PMC5824320

[4] Uccella S. Molecular classification of gastrointestinal and pancreatic neuroendocrine neoplasms: are we ready for that? Endocr Pathol. 2024;35:91-106. 10.1007/s12022-024-09807-2 38470548 PMC11176254

[5] van Riet J , van de Werken HJG, Cuppen E, et al The genomic landscape of 85 advanced neuroendocrine neoplasms reveals subtype-heterogeneity and potential therapeutic targets. Nat Commun. 2021;12:4612. 10.1038/s41467-021-24812-3 34326338 PMC8322054

[6] Girardi DM , Silva ACB, Rêgo JFM, et al Unraveling molecular pathways of poorly differentiated neuroendocrine carcinomas of the gastroenteropancreatic system: a systematic review. Cancer Treat Rev. 2017;56:28-35. 10.1016/j.ctrv.2017.04.002 28456055

[7] Yachida S , Totoki Y, Noë M, et al Comprehensive genomic profiling of neuroendocrine carcinomas of the gastrointestinal system. Cancer Discov. 2022;12:692-711. 10.1158/2159-8290.CD-21-0669 34880079 PMC9394397

[8] Gerard L , Garcia J, Gauthier A, et al ctDNA in neuroendocrine carcinoma of gastroenteropancreatic origin or of unknown primary: the CIRCAN-NEC Pilot Study. Neuroendocrinology. 2021;111:951-964. 10.1159/000512502 33099543

[9] Chen L , Liu M, Zhang Y, et al Genetic characteristics of colorectal neuroendocrine carcinoma: more similar to colorectal adenocarcinoma. Clin Colorectal Cancer. 2021;20:177-185.e13. 10.1016/j.clcc.2020.09.001 33041225

[10] Ooki A , Osumi H, Fukuda K, et al Potent molecular-targeted therapies for gastro-entero-pancreatic neuroendocrine carcinoma. Cancer Metastasis Rev. 2023;42:1021-1054. 10.1007/s10555-023-10121-2 37422534 PMC10584733

[11] Wu H , Yu Z, Liu Y, et al Genomic characterization reveals distinct mutation landscapes and therapeutic implications in neuroendocrine carcinomas of the gastrointestinal tract. Cancer Commun (Lond). 2022;42:1367-1386. 10.1002/cac2.12372 36264285 PMC9759768

[12] Gonzalez RS , Raza A, Propst R, et al Recent advances in digestive tract tumors updates from the 5th edition of the World Health Organization “Blue Book”. Arch Pathol Lab Med. 2021;145:607-626. 10.5858/arpa.2020-0047-RA 32886739

[13] Rindi G , Mete O, Uccella S, et al Overview of the 2022 WHO classification of neuroendocrine neoplasms. Endocr Pathol. 2022;33:115-154. 10.1007/s12022-022-09708-2 35294740

[14] Venizelos A , Elvebakken H, Perren A, et al The molecular characteristics of high-grade gastroenteropancreatic neuroendocrine neoplasms. Endocr Relat Cancer. 2021;29:1-14. 10.1530/ERC-21-0152 34647903 PMC8630776

[15] Martincorena I , Raine KM, Gerstung M, et al Universal patterns of selection in cancer and somatic tissues. Cell. 2017;171:1029-1041 e21. 10.1016/j.cell.2017.09.042 29056346 PMC5720395

[16] Aacr Project G, Aacr Project GENIE: Powering precision medicine through an international consortium. *Cancer Discov*. 2017;7:818-831. 10.1158/2159-8290.CD-17-0151 28572459 PMC5611790

[17] Dowlati A , Lipka MB, McColl K, et al Clinical correlation of extensive-stage small-cell lung cancer genomics. Ann Oncol. 2016;27:642-647. 10.1093/annonc/mdw005 26802149 PMC4803453

[18] Saghaeiannejad Esfahani H , Vela CM, Chauhan A. Prevalence of TP-53/Rb-1 co-mutation in large cell neuroendocrine carcinoma. Front Oncol. 2021;11:653153. 10.3389/fonc.2021.653153 34141612 PMC8203494

[19] Zhuo M , Guan Y, Yang X, et al The prognostic and therapeutic role of genomic subtyping by sequencing tumor or Cell-Free DNA in pulmonary Large-Cell neuroendocrine carcinoma. Clin Cancer Res. 2020;26:892-901. 10.1158/1078-0432.CCR-19-0556 31694833 PMC7024651

[20] Derks J , Leblay N, van Suylen RJ, et al PALGA Group Coauthors Genetic subtypes of large cell neuroendocrine carcinoma (LCNEC) to predict response to chemotherapy. JCO. 2017;35:9061. 10.1200/JCO.2017.35.15_suppl.9061

[21] Park JW , Lee JK, Sheu KM, et al Reprogramming normal human epithelial tissues to a common, lethal neuroendocrine cancer lineage. Science. 2018;362:91-95. 10.1126/science.aat5749 30287662 PMC6414229

[22] Wu H , Yu Z, Liu Y, et al Genomic characterization reveals distinct mutation landscapes and therapeutic implications in neuroendocrine carcinomas of the gastrointestinal tract. Cancer Commun. 2022;42:1367-1386. 10.1002/cac2.12372 PMC9759768 36264285

[23] Kawasaki K , Toshimitsu K, Matano M, et al An organoid biobank of neuroendocrine neoplasms enables Genotype-Phenotype mapping. Cell. 2020;183:1420-1435. 10.1016/j.cell.2020.10.023 33159857

[24] Wiesweg M , Kasper S, Worm K, et al Impact of RAS mutation subtype on clinical outcome-a cross-entity comparison of patients with advanced non-small cell lung cancer and colorectal cancer. Oncogene. 2019;38:2953-2966. 10.1038/s41388-018-0634-0 30568222

[25] Janssen K-P , Alberici P, Fsihi H, et al APC and oncogenic KRAS are synergistic in enhancing wnt signaling in intestinal tumor formation and progression. Gastroenterology. 2006;131:1096-1109. 10.1053/j.gastro.2006.08.011 17030180

[26] Rekhtman N , Pietanza MC, Hellmann MD, et al Next-generation sequencing of pulmonary large cell neuroendocrine carcinoma reveals small cell carcinoma-like and non-small cell carcinoma-like subsets. Clin Cancer Res. 2016;22:3618-3629. 10.1158/1078-0432.CCR-15-2946 26960398 PMC4995776

[27] Rekhtman N , Pietanza CM, Sabari J, et al Pulmonary large cell neuroendocrine carcinoma with adenocarcinoma-like features: Napsin A expression and genomic alterations. Mod Pathol. 2018;31:111-121. 10.1038/modpathol.2017.110 28884744 PMC5937126

[28] Hiroshima K , Iyoda A, Shida T, et al Distinction of pulmonary large cell neuroendocrine carcinoma from small cell lung carcinoma: a morphological, immunohistochemical, and molecular analysis. Mod Pathol. 2006;19:1358-1368. 10.1038/modpathol.3800659 16862075

[29] Hijioka S , Hosoda W, Matsuo K, et al Rb loss and KRAS mutation are predictors of the response to platinum-based chemotherapy in pancreatic neuroendocrine neoplasm with grade 3: a japanese multicenter pancreatic NEN-G3 study. Clin Cancer Res. 2017;23:4625-4632. 10.1158/1078-0432.CCR-16-3135 28455360

[30] Lacombe C , De Rycke O, Couvelard A, et al Biomarkers of response to Etoposide\u2011Platinum chemotherapy in patients with Grade 3 Neuroendocrine Neoplasms. Cancers. 2021;13:643. 10.3390/cancers13040643 33562726 PMC7915900

[31] Hadoux J , Kanaan C, Durand A, et al Prognostic factors of metastatic neuroendocrine carcinoma under first-line treatment with platinum etoposide with a focus on NEC score and Rb expression: Results from the multicentre RBNEC study of the groupe d‘Etude des tumeurs endocrines (GTE) and the ENDOCAN-RENATEN network. Eur J Cancer. 2021;152:100-115. 10.1016/j.ejca.2021.04.030 34090142

[32] Hadoux J , Walter T, Kanaan C, et al ENDOCAN-RENATEN Network. Second-line treatment and prognostic factors in neuroendocrine carcinoma: the RBNEC study. Endocr Relat Cancer. 2022;29:569-580. 10.1530/ERC-22-0102 35920609

[33] Stelwagen J , de Vries EGE, Walenkamp AME. Current treatment strategies and future directions for extrapulmonary neuroendocrine carcinomas a review. JAMA Oncol. 2021;7:759-770. 10.1001/jamaoncol.2020.8072 33630040

[34] Dizdar L , Werner TA, Drusenheimer JC, et al BRAF V600E mutation: a promising target in colorectal neuroendocrine carcinoma. Int J Cancer. 2019;144:1379-1390. 10.1002/ijc.31828 30144031

[35] Jin X-F , Spöttl G, Maurer J, Nölting S, Auernhammer CJ. Antitumoral activity of the MEK inhibitor Trametinib (TMT212) alone and in combination with the CDK4/6 inhibitor Ribociclib (LEE011) in Neuroendocrine Tumor Cells in vitro. Cancers. 2021;13:1485. 10.3390/cancers13061485 33807122 PMC8004919

[36] Owaki S , Mori Y, Nakai S, et al BRAF V600E-mutated colorectal neuroendocrine carcinoma effectively treated with a chemotherapy protocol for BRAF-mutated metastatic colorectal cancer. Intern Med. 2024;63:1995-1999. 10.2169/internalmedicine.2870-23 37981300 PMC11309870

[37] Priestley P , Baber J, Lolkema MP, et al Pan-cancer whole-genome analyses of metastatic solid tumours. Nature. 2019;575:210-216. 10.1038/s41586-019-1689-y 31645765 PMC6872491

[38] Aredo JV , Padda SK, Kunder CA, et al Impact of KRAS mutation subtype and concurrent pathogenic mutations on non-small cell lung cancer outcomes. Lung Cancer. 2019;133:144-150. 10.1016/j.lungcan.2019.05.015 31200821 PMC9348589

[39] Goussia AC , Papoudou-Bai A, Charchanti A, et al Alterations of p53 and Rb pathways are associated with high proliferation in bladder urothelial carcinomas. Anticancer Res. 2018;38:3985-3988. 10.21873/anticanres.12685 29970521

[40] Teo MY , Seier K, Ostrovnaya I, et al Alterations in DNA damage response and repair genes as potential marker of clinical benefit from PD-1/PD-L1 blockade in advanced urothelial cancers. J Clin Oncol. 2018;36:1685-1694. 10.1200/JCO.2017.75.7740 29489427 PMC6366295

[41] Lu M , Zhang P, Zhang Y, et al Efficacy, safety, and biomarkers of toripalimab in patients with recurrent or metastatic neuroendocrine neoplasms: a multiple-center phase ib trial. Clin Cancer Res. 2020;26:2337-2345. 10.1158/1078-0432.CCR-19-4000 32086343

[42] Okamura R , Kato S, Lee S, et al Arid1a alterations function as a biomarker for longer progression\u2011free survival after anti\u2011PD\u20111/PD\u2011L1 immunotherapy. J Immunother Cancer. 2020;8:e000438. 10.1136/jitc-2019-000438 32111729 PMC7057434

