# Supplementary material for: Prognostic and therapeutic roles of specific genotypes through target-gene sequencing on gastroenteropancreatic neuroendocrine carcinoma
Source: Oncologist. 2026 May 8;31(6):oyag185. doi: 10.1093/oncolo/oyag185 (PMC13215382; doi:10.1093/oncolo/oyag185)
Supplement: oyag185_Supplementary_Data [file oyag185_supplementary_data.zip › Supplement Figure Legend.docx]

# Legends of Figure Supplements

# FigureS1 Flowchart of the whole study


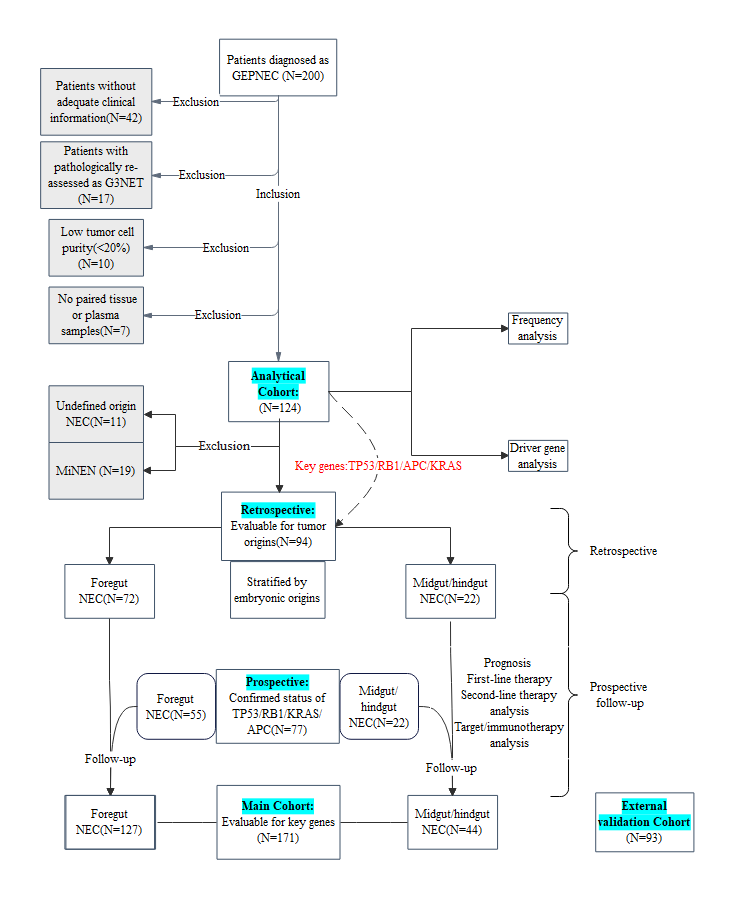


# FigureS2. Oncoprint of ctDNA landscape of GEPNEC.


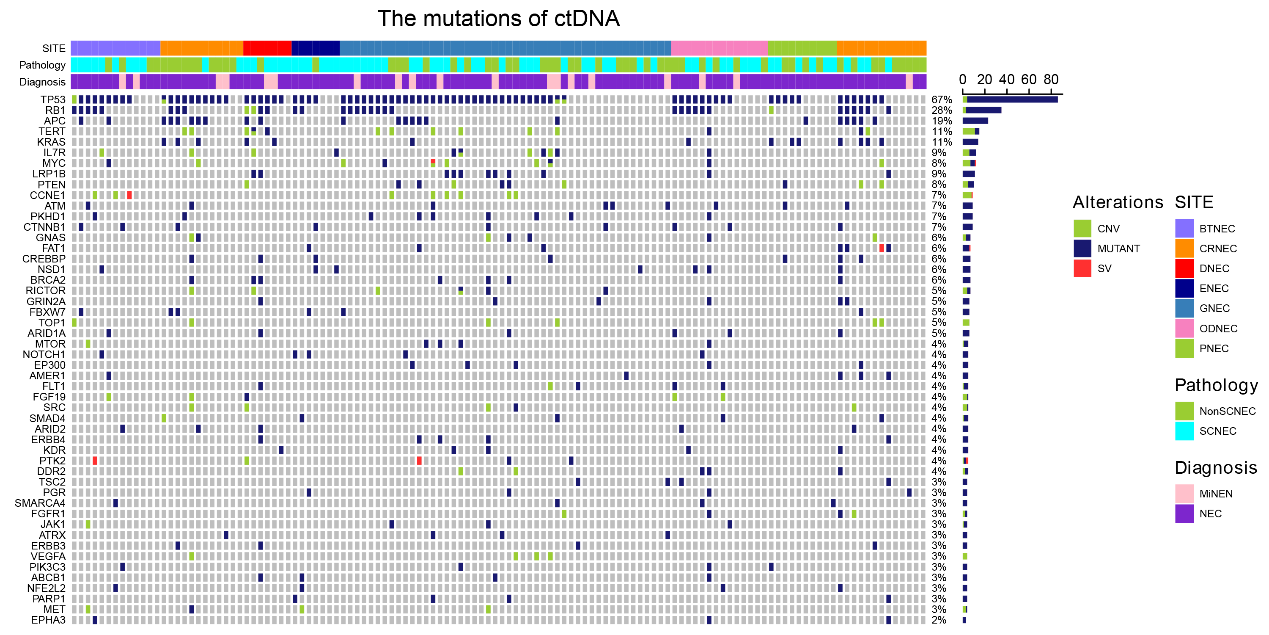


# FigureS3. Concordance of tumor DNA and ctDNA.





# FigureS4. Top-frequency alteration genes in previous report and current study.


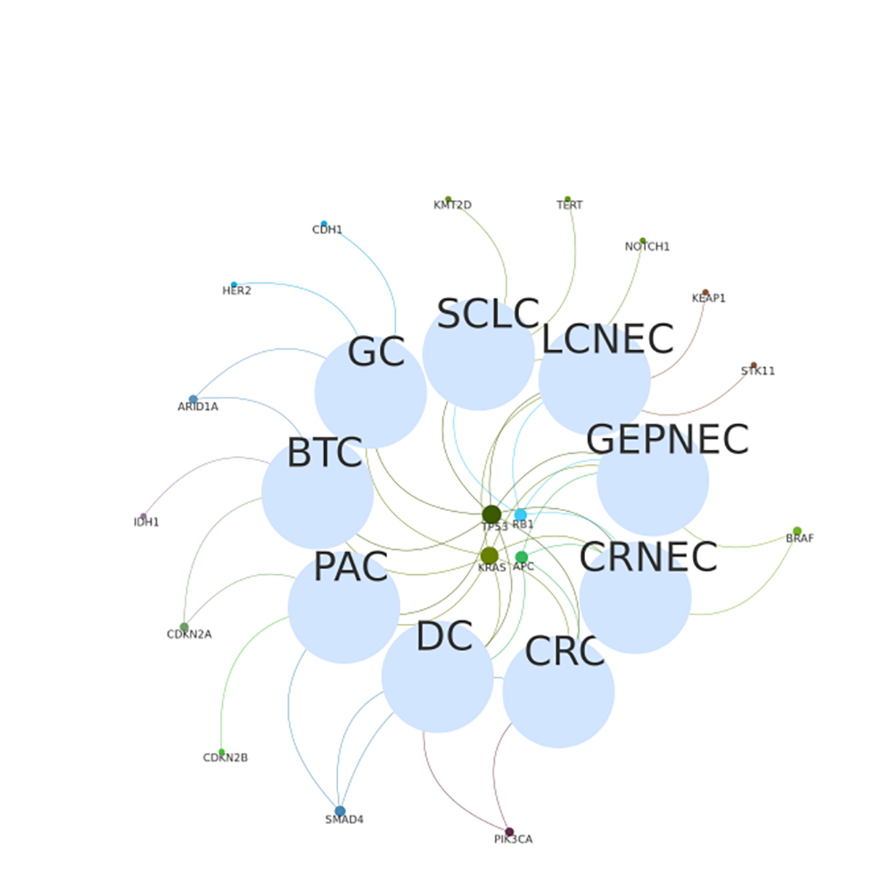


Venn-network plot of high-frequently mutated genes of patients revealed TP53/RB1/KRAS/APC pattern.

# FigureS5. Kaplan-Meier plots of OS.


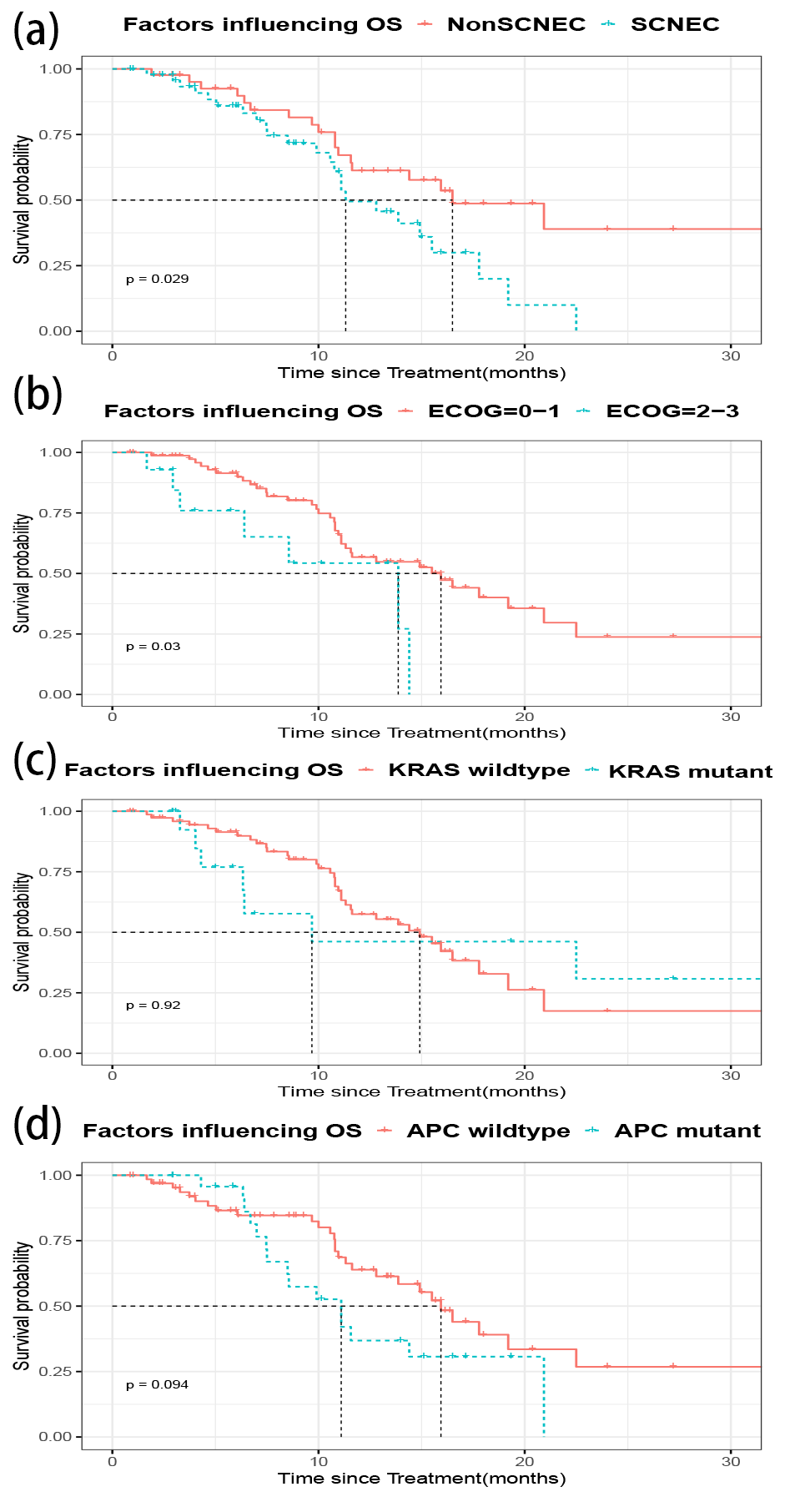


Kaplan-Meier analysis of traditional factors, like (a)pathology, (b)ECOG, and genomic factors, like (c)KRAS status and (d)APC status.

**Figure S6. Kaplan–Meier plot of OS in external validation.**


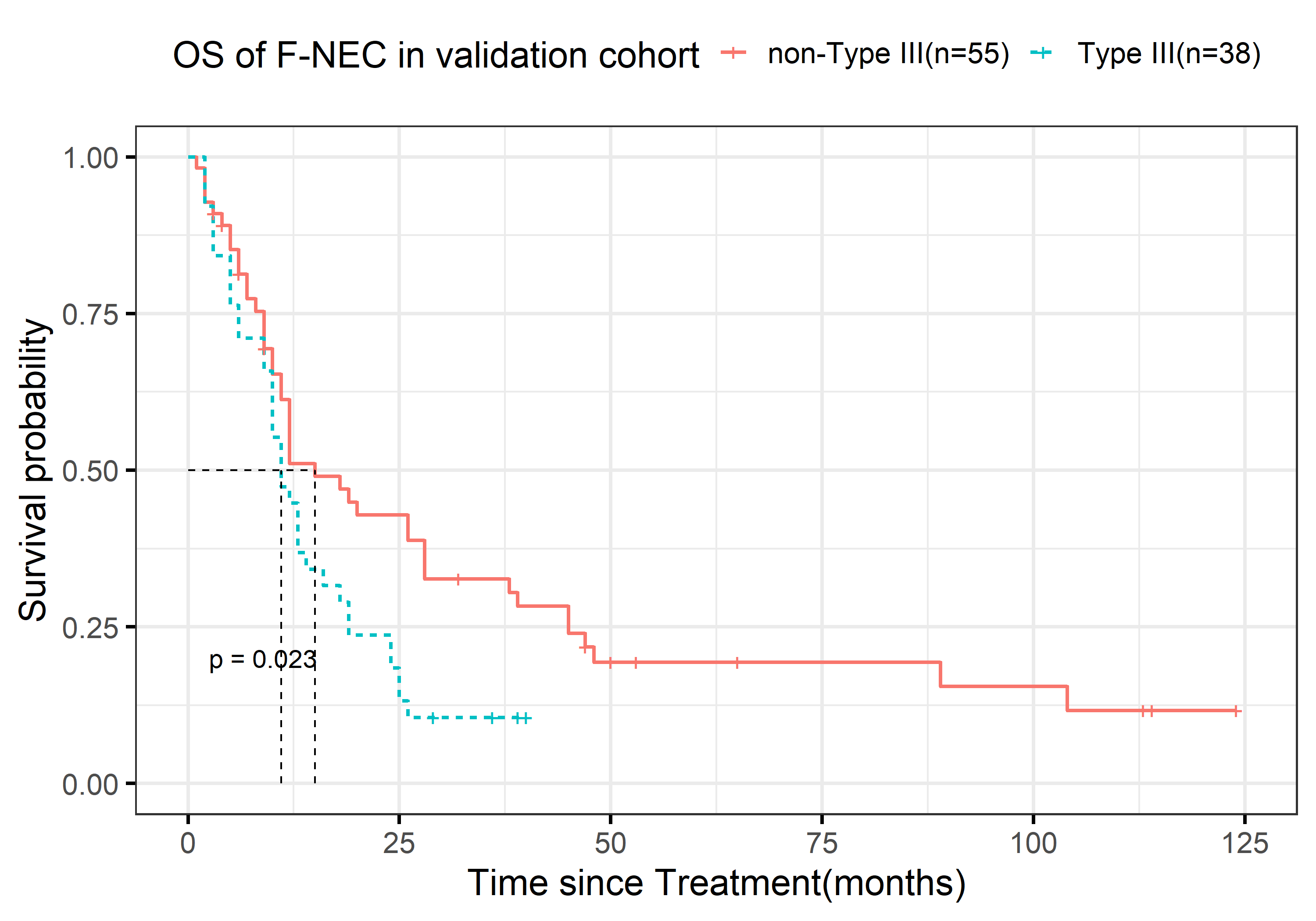


**Figure S7. Kaplan–Meier survival curves of OS by Type-I in GEPNEC**


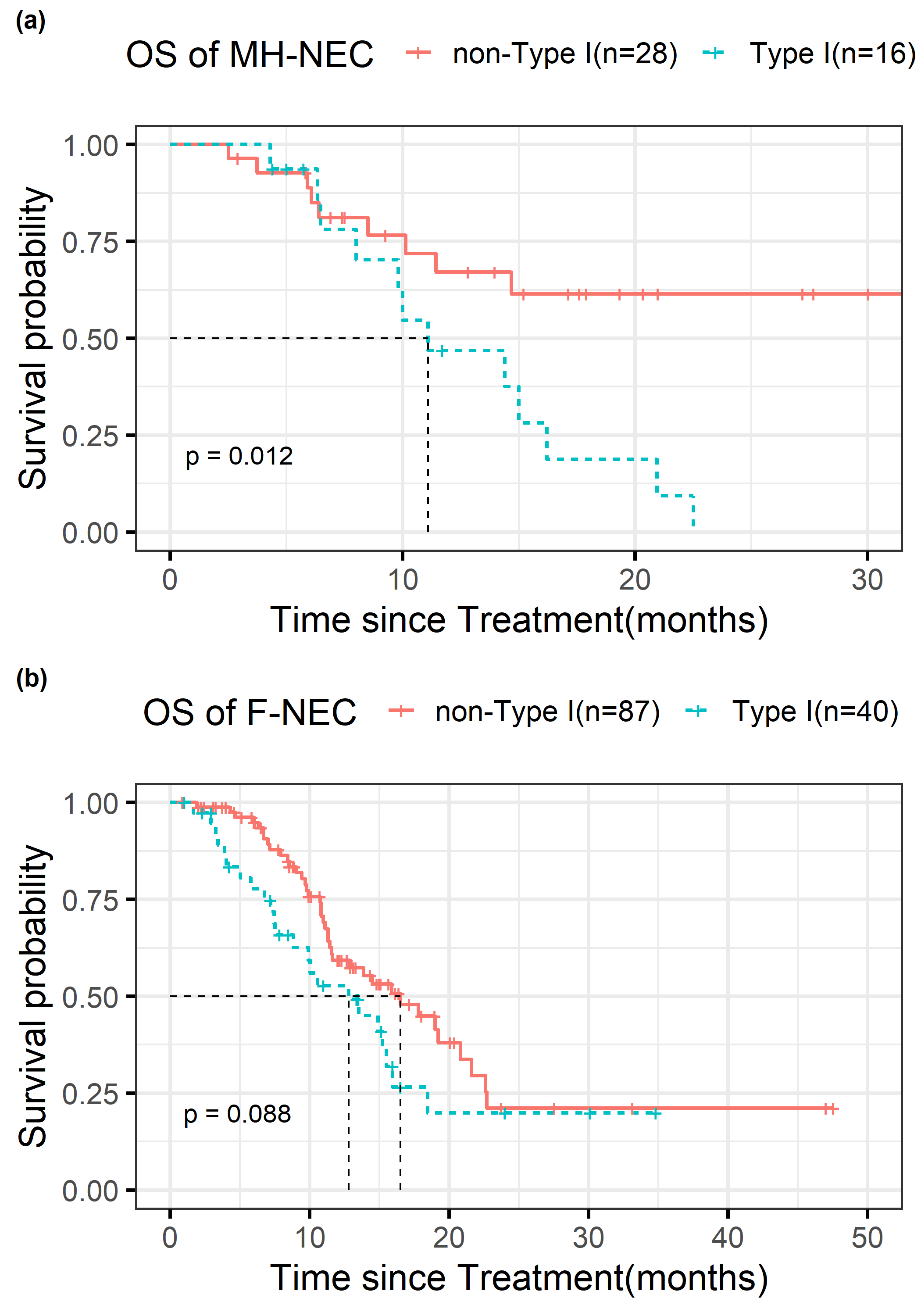
Kaplan-Meier plot of overall survival of Type I on MH-NEC patients (a) and on F-NEC (b).
