# Supplementary material for: Prognostic and therapeutic roles of specific genotypes through target-gene sequencing on gastroenteropancreatic neuroendocrine carcinoma
Source: Oncologist. 2026 May 8;31(6):oyag185. doi: 10.1093/oncolo/oyag185 (PMC13215382; doi:10.1093/oncolo/oyag185)
Supplement: oyag185_Supplementary_Data [file oyag185_supplementary_data.zip › Table Supplement.docx]

# Supplement

# TableS1. Baseline information for the analytical cohort.

|  | level | Overall(n=124) |
| --- | --- | --- |
| Primary site (%) | Esophagus | 7 (5.6) |
|  | Bile duct | 13 (10.5) |
|  | Other | 13 (10.5) |
|  | Stomach | 48 (38.7) |
|  | Duodenum | 7 (5.6) |
|  | Colon | 12 (9.7) |
|  | Rectum | 13 (10.5) |
|  | Pancreas | 10 (8.1) |
|  | Liver | 1 (0.8) |
| Site of Origins (%) | Foregut | 86 (69.4) |
|  | Midgut/hindgut | 25 (20.2) |
|  | Other/Unknown | 13 (10.5) |
| Pathology (%)^a^ | Non-SCNEC | 57 (46.0) |
|  | SCNEC | 67 (54.0) |
| Diagnosis (%) | NEC | 95 (84.7) |
|  | MiNEN | 19 (15.3) |
| Gender (%) | Female | 46 (37.1) |
|  | Male | 78 (62.9) |
| Age (mean (SD)) | | 60.95 (11.00) |
| Ki67 (mean (SD)) | | 0.74 (0.15) |
| Ki67 (%) | ≤55% | 13 (10.5) |
|  | >55 | 111 (89.5) |
| Syn (%)^b^ | - | 4 (5.4) |
|  | + | 70 (94.6) |
| CgA (%)^b^ | - | 15 (26.8) |
|  | + | 41 (73.2) |
| Hepatic Metastases (%) | No | 31 (25.0) |
|  | Yes | 93 (75.0) |
| Lung Metastases (%) | No | 109 (87.9) |
|  | Yes | 15 (12.1) |
| Bone Metastases (%) | No | 113 (91.1) |
|  | Yes | 11 (8.9) |
| NSE (%) | Normal | 40 (39.6) |
|  | Elevated | 61 (60.4) |
| CA199 (%) | Normal | 67 (75.3) |
|  | Elevated | 22 (24.7) |
| CEA (%) | Normal | 68 (77.3) |
|  | Elevated | 20 (22.7) |
| Surgery history (%) | No | 57 (46.0) |
|  | Yes | 67 (54.0) |
| Firstline treatment (%)^c^ | Chemotherapy | 14 (13.5) |
|  | EP | 74 (71.2) |
|  | IP | 14 (13.5) |
|  | Other | 2 (1.9) |
| Firstline ORR (%)^c^ | Non-response | 75 (72.1) |
|  | Response | 29 (27.9) |
| Second-line treatment (%)^c^ | Chemotherapy | 24 (40.7) |
|  | EP | 3 (5.1) |
|  | Immunotherapy | 24 (40.7) |
|  | IP | 7 (11.9) |
|  | Target therapy | 1 (1.7) |
| Second-line ORR (%)^c^ | Non-response | 43 (72.9) |
|  | Response | 16 (27.1) |
| Tumor TMB(%)^e^ | High | 21 (17.2) |
|  | Low | 101 (82.8) |
| Blood TMB(%)^e^ | High | 18 (15.8) |
|  | Low | 96 (84.2) |
| Microsatellite stability (MS) status ^f^ | MSI | 3 (2.4) |
|  | MSS | 101 (81.5) |
|  | Failed | 20 (16.1) |
| Tumor TMB (mean (SD)) |  | 8.90 (14.10) |
| Blood TMB (mean (SD)) |  | 7.27 (10.84) |

Abbreviations: Non-SCNEC, Non-small cell neuroendocrine carcinoma; SCNEC, small cell neuroendocrinal carcinoma. TMB: tumor mutation burden. MSS: microsatellite stability. MSI= microsatellite instability.

a: Non-SCNEC included LCNEC and those with mixed/unknown pathology.

^b^: The Syn and CgA immunohistochemical result was only captured in 74 and 56 patients.

^c^: The confirmed first-line treatment was administered in 104 patients, and the confirmed second-line treatment was observed in 59 patients.

^d^: Immunotherapy-containing therapy was applied in 27 patients.

^e^: 2 patients and 5 patients had incalculable TMB result in tumor TMB and blood TMB.

^f^: Only tumor samples were able to assess MS status and 20 patients had failed the quality control due to the MS sites.

# TableS2. Summary of previous reports on common genomic alterations in gastroenteropancreatic neuroendocrine carcinomas.

| Information | Numbers | Numbers of MiNEN | Site of origin | Methods | Panel | TP53 | | RB1 | | KRAS | | APC | |
| --- | --- | --- | --- | --- | --- | --- | --- | --- | --- | --- | --- | --- | --- |
|  |  |  |  |  |  | Mutation | CNV | Mutation | CNV | Mutation | CNV | Mutation | CNV |
| Current Study | 124 | n/a | GEP | NGS | 124 | 89% | 4% | 38% | 4% | 17% | 2% | 24.2% | 3% |
| Venizelos A, 2021[1] | 152 | n/a | GEP/UNK | NGS | 360 | 63.80% | 0.00% | 10.5%/3.3% | 30.30% | 22.40% | 0.00% | 28% | n/r |
| Puccini A, 2020[2] | 135 | n/a | GEP ^a^ | NGS | 592/44 | 51.00% | n/r | n/r | n/r | 29.40% | n/a | 27% | n/r |
| Sahnane N, 2015[3] | 89 | 36 | GEP | PCR | n/a | n/a | n/a | n/a | n/a | 17.00% | n/r | n/a | n/r |
| Busico A, 2020[4] | 39 | - | GEP | NGS | 50 | 59.00% | n/a | 2.60% | n/a | 7.70% | n/r | 5% | n/r |
| Gerard L, 2021[5] | 24 | - | GEP/UNK | NGS | 78 | 87.50% | n/a | 8.30% | n/a | 12.50% | n/r | 8% | n/r |
| Vijayvergia N, 2016[6] | 23 | - | GEP(14)/Others(9) | NGS | 50 | 56.50% | n/a | 4.30% | n/a | 13.00% | n/r | 9% | n/r |
| Olevian DC, 2016[7] | 32 | 18 | Colon–rectum | qPCR | n/a | n/a | n/a | n/a | n/a | 17.20% | n/r | n/a | n/r |
| Takizawa N, 2015[8] | 24 | 4 | Colon–rectum | PCR | n/a | 20.80% | n/r | n/a | n/a | 4.20% | n/r | 4.00% | n/r |
| Lee SM, 2021[9] | 30 | - | Colon–rectum | NGS | 382 | 43.30% | n/r | 20.00% | 26.70% | 53.30% | n/r | 37.00% | n/r |
| Capdevila J, 2020[10] | 25 | - | Colon | NGS | 61 | 84.00% | 0.00% | 0.00% | 0.00% | 48.00% | n/r | 48.00% | n/r |
| Shamir ER, 2019[11] | 24 | - | Colon–rectum/anus | NGS | 479 | 45.90% | n/r | 45.90% | n/r | 29.20% | 0.00% | 50.00% | n/r |
| Woischke C, 2017[12] | 15 | 10 | Colon–rectum | NGS | 50 | 90.00% | n/r | 30.00% | n/r | 60.00% | n/r | 80.00% | 0.00% |
| Konukiewitz B, 2018[13] | 12 | n/a | Pancreas | NGS | 409 | 66.70% | n/r | 8.30% | 8.30% | 41.70% | n/r | 8.00% | n/r |
| Liu F, 2021[14] | 15 | n/a | Gallbladder | WES | - | 73.30% | 0.00% | 26.70% | n/r | n/r | 0.00% | 0.00% | n/r |
| Li R, 2021[15] | 46 | n/a | Esophagus | WES | - | 84.80% | 0.00% | 34.80% | 28.20% | n/r | 0.00% | n/r | 0.00% |
| Meder L, 2016[16] | 26 | n/a | Extra-pulmonary | NGS | n/a | 76.90% | n/r | 46.20% | n/r | n/a | n/r | n/a | n/r |
| Bergsland EK, 2016[17] | 123 | n/a | Pancreas | NGS | 192 | 18.00% | n/r | 10.00% | n/r | 7.00% | n/r | 3.00% | n/r |
| Bergsland EK, 2016[17] | 92 | n/a | Colon | NGS | 192 | 59.00% | n/r | 34.00% | n/r | 37.00% | n/r | 47.00% | n/r |
| Bergsland EK, 2017[17] | 59 | n/a | oesophageal, stomach, small intestine | NGS | 192 | 49.00% | n/r | 29.00% | n/r | 3.00% | n/r | 8.00% | n/r |
| Rie Makuuch 2017[18] | 6 | - | Stomach | NGS | 35 | 100% | n/r | 16.70% | n/r | 16.70% | n/r | 0.00% | n/r |
| Maria Scardoni2014 [19] | 6 | - | Stomach | Sanger | - | 83.33% | n/r | 16.70% | n/r | 16.70% | n/r | 0.00% | n/r |
| Jiwon Koh2021[20] | 13 | 6 | Stomach | WES | - | 66.70% | n/r | 7.70% | n/r | 7.70% | n/r | 23.10% | n/r |

Abbreviations: CNV, copy-number variation analysis; GEP, gastroenteropancreatic tract; NGS, next-generation sequencing; n/r, gene not reported or applicable; PCR, polymerase chain reaction; WES, whole-exome sequencing; WGS, whole-genome sequencing.

Note: if a range is provided, n varied across genes analyzed based on tissue availability).

a. This includes both neuroendocrine carcinomas and G3 well-differentiated neuroendocrine tumors; however, relative proportions of the two subgroups are not provided.

# TableS3. The dNdScv algorithm analysis of driver genes in GEPNEC

| **Symbol** | **Alteration numbers^b^** | **n_mis** | **n_non** | **n_spl** | **n_ind** | | **CNV** | | **pglobal_cv** | **qglobal_cv** |
| --- | --- | --- | --- | --- | --- | --- | --- | --- | --- | --- |
| TP53 | 94 | 68 | 13 | 8 | 0 | 5 | | <0.01 | | <0.01 |
| APC | 49 | 12 | 25 | 1 | 7 | 4 | | <0.01 | | <0.01 |
| RB1 | 37 | 5 | 15 | 12 | 0 | 5 | | 0.025 | | 0.040 |
| KRAS | 23 | 21 | 0 | 0 | 0 | 2 | | <0.01 | | <0.01 |
| CCNE1 | 21 | 4 | 1 | 1 | 0 | 15 | | 0.010 | | 0.018 |
| LRP1B | 21 | 21 | 2 | 1 | 0 | 0 | | <0.01 | | <0.01 |
| IL7R | 16 | 5 | 1 | 1 | 0 | 9 | | 0.017 | | 0.028 |
| TERT | 15 | 2 | 0 | 1 | 0 | 12 | | <0.01 | | <0.01 |
| ARID1A | 12 | 4 | 2 | 3 | 3 | 0 | | <0.01 | | <0.01 |
| RICTOR | 12 | 4 | 0 | 0 | 0 | 8 | | 0.109 | | 0.151 |
| BRCA2 | 11 | 10 | 0 | 0 | 1 | 0 | | <0.01 | | <0.01 |
| CTNNB1 | 11 | 10 | 0 | 1 | 0 | 0 | | 0.515 | | 0.564 |
| NOTCH1 | 10 | 10 | 0 | 0 | 0 | 0 | | <0.01 | | <0.01 |
| EPHA3 | 10 | 7 | 2 | 1 | 0 | 0 | | 0.865 | | 0.872 |
| SMARCA4 | 9 | 7 | 2 | 0 | 0 | 0 | | <0.01 | | <0.01 |
| ATM | 9 | 5 | 2 | 1 | 1 | 0 | | <0.01 | | <0.01 |
| ERBB4 | 9 | 7 | 0 | 1 | 1 | 0 | | <0.01 | | <0.01 |
| MYC | 9 | 4 | 0 | 0 | 0 | 5 | | <0.01 | | <0.01 |
| CREBBP | 8 | 4 | 0 | 1 | 3 | 0 | | <0.01 | | <0.01 |
| MET | 8 | 4 | 0 | 0 | 0 | 4 | | <0.01 | | <0.01 |
| ERBB2 | 8 | 6 | 0 | 0 | 1 | 1 | | <0.01 | | <0.01 |
| SMAD4 | 8 | 5 | 1 | 0 | 0 | 2 | | 0.519 | | 0.564 |
| Foregut NEC | | | | | | | | | | |
| APC | 19 | 5 | 7 | 0 | 3 | 4 | | <0.01 | | <0.01 |
| TP53 | 56 | 44 | 6 | 4 | 0 | 2 | | <0.01 | | <0.01 |
| RB1 | 28 | 4 | 8 | 11 | 0 | 5 | | <0.01 | | <0.01 |
| CCNE1 | 13 | 1 | 0 | 0 | 0 | 12 | | <0.01 | | <0.01 |
| KRAS | 5 | 3 | 1 | 0 | 0 | 2 | | <0.01 | | <0.01 |
| LRP1B | 15 | 13 | 2 | 0 | 0 | 0 | | <0.01 | | <0.01 |
| Midgut/hindgut NEC | | | | | | | | | | |
| APC | 13 | 2 | 11 | 0 | 0 | 0 | | <0.01 | | <0.01 |
| TP53 | 21 | 12 | 3 | 5 | 0 | 0 | | 0.018 | | 0.084 |
| KRAS | 9 | 9 | 0 | 0 | 0 | 0 | | 0.02 | | 0.099 |
| ERBB4 | 3 | 3 | 0 | 0 | 0 | 0 | | 0.03 | | 0.14 |

a: Alteration proportions referred to the proportions of patients with alterations in populations.

b: Alteration numbers referred to the sum of all the alterations.

Abbreviations: n_mis: numbers of missense mutation; n_non: numbers of missense mutation; n_spl: numbers of splicing mutation; n_ind: numbers of insert/deletion; CNV: copy number variations.

# TableS4. Cox proportional hazard models for overall survival of the analytical cohort.

| Univariate analysis | | | |
| --- | --- | --- | --- |
| **Characteristic** | Hazard Ratio | CI95 | P Value |
| Site (Bile duct vs Esophagus) | 0.34 | 0.07-1.55 | 0.162 |
| Site (Others vs Esophagus) | 0.33 | 0.08-1.31 | 0.114 |
| Site (Stomach vs Esophagus) | 0.5 | 0.16-1.49 | 0.213 |
| Site (Duodenum vs Esophagus) | 0.95 | 0.24-3.84 | 0.948 |
| Site (Colon vs Esophagus) | 0.5 | 0.11-2.22 | 0.359 |
| Site (Rectum vs Esophagus) | 0.44 | 0.11-1.78 | 0.249 |
| Site (Pancreas vs Esophagus) | 1.4 | 0.41-4.81 | 0.591 |
| Site (Liver vs Esophagus) | 4.51 | 0.48-42.9 | 0.189 |
| Origin (Midgut/hindgut vs Foregut) | 0.72 | 0.32-1.61 | 0.42 |
| Origin (Others vs Foregut) | 0.56 | 0.21-1.48 | 0.241 |
| Pathology (SCNEC vs Non-SCNEC) | 1.21 | 0.67-2.19 | 0.519 |
| Gender (Female vs Male) | 0.82 | 0.46-1.47 | 0.512 |
| Age | 1.01 | 0.98-1.04 | 0.638 |
| Ki67 | 1.52 | 0.25-9.43 | 0.651 |
| Hepatic Metastases (Yes vs No) | 1.27 | 0.67-2.42 | 0.469 |
| Lung Metastases (Yes vs No) | 0.9 | 0.35-2.28 | 0.818 |
| Bone Metastases (Yes vs No) | 0.54 | 0.21-1.41 | 0.209 |
| NSE (Elevated vs Normal) | 1.69 | 0.77-3.72 | 0.189 |
| CA199 (Elevated vs Normal) | 1.61 | 0.77-3.36 | 0.206 |
| CEA (Elevated vs Normal) | 1.09 | 0.49-2.47 | 0.828 |
| Surgery (Yes vs No) | 1.11 | 0.62-1.97 | 0.735 |
| First-line Response (Yes vs No) | 0.86 | 0.45-1.66 | 0.658 |
| Second-line Response (Yes vs No) | 0.22 | 0.08-0.58 | 0.002 |
| Genomic features | | | |
| tumor TMB (Low vs High) | 2.26 | 0.94-5.39 | 0.067 |
| blood TMB (Low vs High) | 2.46 | 0.86-7.04 | 0.094 |
| tumor TMB (continuous) | 1 | 0.98-1.03 | 0.757 |
| blood TMB (continuous) | 0.97 | 0.94-1.01 | 0.159 |
| TP53_mutant (Yes vs No) | 0.88 | 0.42-1.83 | 0.732 |
| RB1_mutant (Yes vs No) | 1.39 | 0.78-2.46 | 0.266 |
| KRAS_mutant (Yes vs No) | 0.82 | 0.38-1.75 | 0.601 |
| APC_mutant (Yes vs No) | 1.56 | 0.81-3.01 | 0.184 |
| CCNE1_CNV (Yes vs No) | 0.66 | 0.26-1.68 | 0.384 |
| LRP1B_mutant (Yes vs No) | 1.38 | 0.61-3.12 | 0.433 |
| ARID1A_mutant (Yes vs No) | 1.63 | 0.64-4.14 | 0.306 |
| PKHD1 mutant (Yes vs No) | 1.97 | 0.83-4.68 | 0.124 |
| TERT_CNV (Yes vs No) | 1.39 | 0.62-3.12 | 0.423 |
| BRCA2_mutant (Yes vs No) | 0.49 | 0.15-1.58 | 0.234 |
| ATM_mutant (Yes vs No) | 0.42 | 0.06-3.08 | 0.394 |
| ATM_germline (Yes vs No) | 0.25 | 0.03-1.8 | 0.168 |
| NOTCH1_mutant (Yes vs No) | 1.32 | 0.59-2.96 | 0.498 |
| TSC2_germline (Yes vs No) | 0.53 | 0.16-1.73 | 0.295 |
| EPHA3_mutant (Yes vs No) | 6.2 | 1.97-19.53 | 0.002 |
| MYC_CNV (Yes vs No) | 2.3 | 0.82-6.47 | 0.115 |
| CTNNB1_mutant (Yes vs No) | 1.44 | 0.56-3.67 | 0.446 |
| CDKN2A_mutant (Yes vs No) | 0.84 | 0.12-6.13 | 0.865 |
| Pathway Alteration | | | |
| Cell cycle (Yes vs No) | 1.03 | 0.58-1.83 | 0.927 |
| Hippo (Yes vs No) | 1.06 | 0.37-2.99 | 0.915 |
| MYC (Yes vs No) | 2.12 | 0.83-5.45 | 0.117 |
| NOTCH (Yes vs No) | 1.04 | 0.57-1.91 | 0.89 |
| PI3K (Yes vs No) | 1.43 | 0.79-2.6 | 0.241 |
| TGFβ(Yes vs No) | 2.1 | 0.97-4.55 | 0.061 |
| RTK/RAS (Yes vs No) | 1.08 | 0.61-1.92 | 0.789 |
| WNT (Yes vs No) | 1.43 | 0.79-2.59 | 0.243 |
| TP53 related (Yes vs No) | 0.91 | 0.42-1.96 | 0.813 |
| DDR pathway alterations (Yes vs No) | 1.05 | 0.56-1.98 | 0.875 |
| Multivariate analysis | | | |
| Characteristic | Hazard Ratio | CI95 | P Value |
| Blood allele Frequency | 1.01 | 1.00, 1.03 | 0.012 |
| tumor TMB (Low vs High) | 4.8 | 1.54, 15.0 | 0.007 |
| EPHA3_mutant (Yes vs No) | 10.6 | 2.53, 44.0 | 0.001 |
| TGFβ pathway (Yes vs No) | 2.16 | 0.85, 5.48 | 0.11 |
| Abbreviations: HR = Hazard Ratio, CI95 = Confidence Interval. Non-SCNEC, Non-small cell neuroendocrine carcinoma; SCNEC, small cell neuroendocrinal carcinoma.  a: TMB was divided according to the median values as low and high. | | | |

# TableS5. Cox regression screening of key gene-based genotypes.

|  | **MH-NEC** | | | | **F-NEC** | | | |
| --- | --- | --- | --- | --- | --- | --- | --- | --- |
| **Factors (if not denoted, the default is “Yes vs No”)** | **Hazard Ratio** | **CI95** | **P** | **n.** | **Hazard Ratio** | **CI95** | **P** | **n.** |
|  | **Univariate Cox regression analysis** | | | | | | | |
| TP53 alteration | 0.3 | 0.06-1.49 | 0.142 | 23 | 1.03 | 0.39-2.67 | 0.958 | 71 |
| RB1 alteration | 2.39 | 0.59-9.68 | 0.22 | 10 | 1.62 | 0.84-3.13 | 0.153 | 27 |
| KRAS alteration | 80684217 | 0-Inf | 0.999 | 12 | 1.93 | 0.74-5.03 | 0.177 | 9 |
| APC alteration | 2.48 | 0.63-9.76 | 0.193 | 13 | 1.77 | 0.85-3.7 | 0.128 | 16 |
| TP53/RB1co-alteration(Type I) | 2.39 | 0.43-13.2 | 0.319 | 9 | 1.72 | 0.87-3.38 | 0.113 | 27 |
| TP53/RB1co-alteration+KRAS_alteration+APC_wild-type | 0.55 | 0.06-4.95 | 0.594 | 1 | 3.31 | 1-10.97 | 0.05 | 3 |
| TP53/RB1co-alteration+KRAS_wild-type+APC_alteration | 1.45 | 0.35-6.04 | 0.611 | 3 | 1.83 | 0.64-5.2 | 0.257 | 5 |
| TP53/RB1co-alteration+KRAS/APC co-alteration | 24.89 | 2.15-287.84 | 0.01 | 3 | 0 | 0-Inf | 0.998 | 1 |
| TP53/RB1co-alteration+KRAS/APC co-wild-type | 2.54 | 0.29-21.87 | 0.396 | 2 | 1.05 | 0.48-2.31 | 0.896 | 18 |
| TP53/RB1co-wild-type+KRAS/APC co-alteration | 0 | 0-Inf | 0.999 | 1 | 2.09 | 0.85-5.1 | 0.107 | 1 |
| TP53/RB1co-wild-type+KRAS_alteration+APC_wild-type | - | - | - | 0 | - | - | - | 0 |
| TP53/RB1co-wild-type+KRAS_wild-type+APC_alteration | - | - | - | 0 | 0 | 0-Inf | 0.997 | 1 |
| TP53/RB1co-wild-type+KRAS/APC_co-wild-type | - | - | - | 0 | 0.45 | 0.23-0.89 | 0.021 | 14 |
| TP53alteration+RB1wild-type+KRAS/APC_co-alteration | 0 | 0-Inf | 0.999 | 3 | 0 | 0-Inf | 0.997 | 1 |
| TP53alteration+RB1wild-type+KRAS_alteration+APC_wild-type | 0 | 0-Inf | 0.999 | 4 | 2.43 | 0.56-10.6 | 0.237 | 4 |
| TP53alteration+RB1wild-type+KRAS_wild-type+APC_alteration | 0.9 | 0.1-7.73 | 0.922 | 3 | 1.8 | 0.69-4.71 | 0.228 | 8 |
| TP53alteration+RB1wild-type+KRAS/APC_co-wild-type | 6.27 | 0.88-44.73 | 0.067 | 4 | 0.45 | 0.21-0.97 | 0.04 | 31 |
| TP53wild-type+RB1alteration+KRAS_alteration+APC_wild-type | - | - | - | 0 | - | - | - | 0 |
| TP53wild-type+RB1alteration+KRAS_wild-type+APC_alteration | - | - | - | 0 | - | - | - | 0 |
| TP53wild-type+RB1alteration+KRAS/APC_co-alteration | - | - | - | 0 | - | - | - | 0 |
| TP53wild-type+RB1alteration+KRAS/APC_co-wild-type | 0 | 0-Inf | 0.998 | 1 | - | - | - | 0 |
| TP53/RB1co-wild-type+No KRAS/APC_co-alteration | 0.43 | 0.11-1.68 | 0.222 | 12 | 0.69 | 0.36-1.34 | 0.278 | 58 |
| TP53/RB1co-wild-type+KRAS/APC either alteration | 0.23 | 0.22-1.24 | 0.105 | 11 | 1.81 | 0.81-4.03 | 0.148 | 14 |
| TP53/RB1co-alteration+KRAS/APC either alteration | 2.25 | 0.6-8.37 | 0.228 | 7 | 2.38 | 1.04-5.47 | 0.04 | 9 |
| TP53/RB1co-alteration+No KRAS/APC_co-alteration | 1.32 | 0.36-4.81 | 0.67 | 6 | 1.62 | 0.84-3.14 | 0.149 | 26 |
| TP53/RB1 either alteration+KRAS/APC either alteration | 0.39 | 0.08-1.94 | 0.248 | 17 | 2.43 | 0.56-10.6 | 0.237 | 22 |
| TP53/RB1 either alteration+No KRAS/APC co-alteration | 0.25 | 0.04-1.4 | 0.114 | 18 | 1.26 | 0.49-3.26 | 0.637 | 69 |
| TP53/RB1 either alteration+KRAS/APC co-alteration | 22.96 | 0.92-274.76 | 0.053 | 6 | 0 | 0-Inf | 0.997 | 2 |
| TP53/RB1 either alteration+KRAS/APC co-wild-type | 3.84 | 0.74-19.94 | 0.109 | 7 | 0.5 | 0.25-0.98 | 0.044 | 49 |
| No TP53/RB1co-alteration+KRAS/APC either alteration | 0.13 | 0.02-1.04 | 0.044 | 11 | 1.81 | 0.81-4.03 | 0.148 | 14 |
| No TP53/RB1co-alteration+No KRAS/APC co-alteration | 3.33 | 0.54-20.61 | 0.196 | 12 | 0.69 | 0.36-1.34 | 0.278 | 58 |
| No TP53/RB1co-alteration+KRAS/APC co-alteration | 0 | 0-Inf | 0.999 | 4 | 0 | 0-Inf | 0.997 | 1 |
| No TP53/RB1co-alteration+KRAS/APC co-wild-type† | 3.33 | 0.54-20.61 | 0.196 | 5 | 0.45 | 0.23-0.89 | 0.021 | 45 |
| TP53/RB1co-wild-type or No KRAS/APC co-alteration | 0.04 | 0-0.46 | 0.01 | 22 | 1227148 | 0-Inf | 0.998 | 85 |
| TP53/RB1co-wild-type or KRAS/APC either alteration | 0.39 | 0.05-3.39 | 0.396 | 23 | 0.95 | 0.43-2.08 | 0.896 | 68 |
| TP53/RB1co-alteration or KRAS/APC either alteration† | 0.3 | 0.05-1.86 | 0.196 | 20 | 2.21 | 1.12-4.36 | 0.021* | 41 |
| TP53/RB1co-alteration or No KRAS/APC co-alteration | 74482376 | 0-Inf | 0.999 | 21 | 25599861 | 0-Inf | 0.997 | 95 |
| TP53/RB1 either alteration or KRAS/APC either alteration |  | NA-NA |  | 25 | 1.26 | 0.44-3.6 | 0.663 | 72 |
| TP53/RB1 either alteration or No KRAS/APC co-alteration | 73595452 | 0-Inf | 0.999 | 24 |  | NA-NA |  | 86 |
| TP53/RB1 either alteration or KRAS/APC co-alteration |  | NA-NA |  | 25 | 1.03 | 0.39-2.67 | 0.958 | 71 |
| TP53/RB1 either alteration or KRAS/APC co-wild-type | 73595452 | 0-Inf | 0.999 | 24 | 0.21 | 0.03-1.58 | 0.128 | 85 |
| No TP53/RB1 co-alteration or KRAS/APC either alteration | 0.39 | 0.05-3.39 | 0.396 | 23 | 0.95 | 0.43-2.08 | 0.896 | 68 |
| No TP53/RB1 co-alteration or No KRAS/APC co-alteration | 0.04 | 0-0.46 | 0.01 | 22 | 1227148 | 0-Inf | 0.998 | 85 |
| No TP53/RB1 co-alteration or KRAS/APC co-alteration | 0.76 | 0.21-2.74 | 0.67 | 19 | 0.62 | 0.32-1.19 | 0.149 | 60 |
| No TP53/RB1 co-alteration or KRAS/APC co-wild-type | 0.45 | 0.12-1.66 | 0.228 | 18 | 0.42 | 0.18-0.96 | 0.04 | 77 |
|  | **Multivariate Cox regression analysis** | | | | | | | |
| TP53/RB1 co-alteration or KRAS/APC either alteration | - | - | - | - | 2.2 | 1.02-6.64 | 0.04 | 41 |
| TP53/RB1 either alteration；KRAS/APC co-wild-type | - | - | - | - | 0.55 | 0.23-1.34 | 0.19 | 49 |
| TP53 alteration+RB1wild-type+KRAS/APC co-wild-type | - | - | - | - | 1.25 | 0.29-5.36 | 0.76 | 31 |
| TP53/RB1_co-alteration；KRAS/APC either alteration | 0.33 | 0.02-0.99 | 0.049 | 11 | - | - | - | - |
| Age | 1.01 | 0.98-1.05 | 0.466 | - | 1 | 0.98-1.03 | 0.7 | - |
| Gender(Female vs Male) | 1.21 | 0.44-3.34 | 0.716 | - | 1.07 | 0.63-1.82 | 0.81 | - |

†：The 2 groups were indeed the mutually complemented population. CI95=95% confidential interval

# TableS6. Genotype I-based stratification of main cohort.

| Stratification | | | Factors | Main cohort(n=210) | | | | | | | | |
| --- | --- | --- | --- | --- | --- | --- | --- | --- | --- | --- | --- | --- |
|  | | | | Non-Type-I (n=150) | | Type-I(n=60) | | | | p | | |
| Site (%) | Esophagus | | | 9 (6.0) | 4 (6.7) | | 0.118 | | | |  |  |
|  | Stomach | | | 58 (38.7) | 18 (30.0) | |  | | | |  |  |
|  | Duodenum | | | 5 (3.3) | 7 (11.7) | |  | | | |  |  |
|  | Pancreas | | | 22 (14.7) | 5 (8.3) | |  | | | |  |  |
|  | Liver | | | 2 (1.3) | 0 (0.0) | |  | | | |  |  |
|  | Bile tract | | | 12 (8.0) | 10 (16.7) | |  | | | |  |  |
|  | Colon | | | 18 (12.0) | 6 (10.0) | |  | | | |  |  |
|  | Rectum | | | 24 (16.0) | 10 (16.7) | |  | | | |  |  |
| Age (mean (SD)) | | | | 60.30 (12.15) | 61.17 (9.29) | | 0.62 | | | |  |  |
| Pathology (%) | Non-SCNEC | | | 85 (56.7) | 21 (35.0) | | 0.007 | | | |  |  |
|  | | SCNEC | | 65 (43.3) | 39 (65.0) | |  | | | |  |  |
| Ki67 (mean (SD)) | | | | 0.72 (0.14) | 0.77 (0.13) | | 0.016 | | | |  |  |
| Gender (%) | Female | | | 37 (24.7) | 27 (45.0) | | 0.006 | | | |  |  |
|  | Male | | | 113 (75.3) | 33 (55.0) | |  | | | |  |  |
| Stage (%) | I-III | | | 10 (6.7) | 1 (1.7) | | 0.26 | | | |  |  |
|  | IV | | | 140 (93.3) | 59 (98.3) | |  | | | |  |  |
| Diagnosis (%) | MiNEN | | | 35 (23.3) | 4 (6.7) | | 0.009 | | | |  |  |
|  | NEC | | | 115 (76.7) | 56 (93.3) | |  | | | |  |  |
| Hepatic | No | | | 32 (21.3) | 11 (18.3) | | 0.766 | | | |  |  |
| metastases (%) | Yes | | | 118 (78.7) | 49 (81.7) | |  | | | |  |  |
| Lung | No | | | 131 (87.3) | 48 (80.0) | | 0.255 | | | |  |  |
| metastases (%) | Yes | | | 19 (12.7) | 12 (20.0) | |  | | | |  |  |
| Bone | No | | | 139 (92.7) | 53 (88.3) | | 0.459 | | | |  |  |
| metastases (%) | Yes | | | 11 (7.3) | 7 (11.7) | |  | | | |  |  |
| NSE (%)^$^ | No | | | 66 (50.4) | 14 (25.5) | | 0.003 | | | |  |  |
|  | Elevated | | | 65 (49.6) | 41 (74.5) | |  | | | |  |  |
| CA199 (%)^$^ | No | | | 86 (72.6) | 40 (76.9) | | 0.693 | | | |  |  |
|  | Elevated | | | 33 (27.4) | 12 (23.1) | |  | | | |  |  |
| CEA (%)^$^ | No | | | 78 (66.7) | 39 (75.0) | | 0.367 | | | |  |  |
|  | Elevated | | | 39 (33.3) | 13 (25.0) | |  | | | |  |  |
| Smoking history (%)^‖^ | No | | | 35 (64.8) | 80 (64.5) | | | | 0.956 | | |  |
|  | Yes | | | 19 (35.2) | 44 (35.5) | | | |  | | |  |
| Surgery history (%) | No | | | 75 (50.0) | 29 (48.3) | | | | 0.948 | | |  |
|  | Yes | | | 75 (50.0) | 31 (51.7) | | | |  | | |  |
| TMB (%) | High | | | 23 (15.3) | 6 (10.0) | | | | 0.429 | | |  |
|  | Low | | | 127 (84.7) | 54 (90.0) | | | |  | | |  |
| First-line (%)* | - | | | 9 (6.0) | 1 (1.7) | | | | 0.065 | | |  |
|  | EP | | | 83 (55.3) | 43 (71.7) | | | |  | | |  |
|  | IP | | | 16 (10.7) | 8 (13.3) | | | |  | | |  |
|  | CAPTEM | | | 2 (1.3) | 0 (0.0) | | | |  | | |  |
|  | Other Chemotherapy | | | 39 (26.0) | 6 (10.0) | | | |  | | |  |
|  | Other Treatment | | | 1 (0.7) | 2 (3.3) | | | |  | | |  |
| Second-line (%)· | EP | | | 10 (9.6) | 3 (7.5) | | | | 0.165 | | |  |
|  | IP | | | 4 (3.8) | 7 (17.5) | | | |  | | |  |
|  | CAPTEM | | | 2 (1.9) | 1 (2.5) | | | |  | | |  |
|  | Other Chemotherapy | | | 50 (48.1) | 15 (37.5) | | | |  | | |  |
|  | Immunotherapy | | | 32 (30.8) | 13 (32.5) | | |  | | | |  |
|  | Target therapy | | | 3 (2.9) | 0 (0.0) | | |  | | | |  |
|  | Other Treatment | | | 3 (2.9) | 1 (2.5) | | |  | | | |  |
| First-line efficacy (%)§ | Response | | | 90 (68.7) | 35 (62.5) | | | 0.512 | | | |  |
|  | Non-response | | | 41 (31.3) | 21 (37.5) | | |  | | | |  |
| Second-line efficacy (%)† | Response | | | 75 (78.9) | 28 (73.7) | | | 0.67 | | | |  |
|  | Non-response | | | 20 (21.1) | 10 (26.3) | | |  | | | |  |

The Table 1 and Table.S4 had same annotation. $：26 patients had no baseline NSE level. *: -: Only adjuvant chemotherapy. Other CT: Other chemotherapy, Other Trt: other treatment, including radiotherapy, intervention and other local treatment. · ：There were 49 patients in F-NEC with no second-line information and 17 patients had no second-line information in MH-NEC. §：There were 17 patients in F-NEC had no confirmed evaluation，and the number was 6 in MH-NEC. †：There were 55 patients in F-NEC had no confirmed evaluation，and the number was 22 in MH-NEC.‖: There were 32 patients had no confirmed smoking history.

# TableS7. Univariate Cox proportional hazard regression analysis on Type II GEPNEC receiving first-line therapy

| **Factors** | **Hazard Ratio** | | **CI95** ^a^ | **P Value** |
| --- | --- | --- | --- | --- |
| Univariate analysis | | | | |
| Site (Rectum vs Colon) | 0.43 | 0.16-1.17 | | 0.097 |
| Gender (Male vs Female) | 1.22 | 0.39-3.81 | | 0.734 |
| Age | 0.96 | 0.9-1.02 | | 0.215 |
| Pathology (SCNEC vs Non-SCNEC) | 2.13 | 0.71-6.39 | | 0.179 |
| Diagnosis (NEC vs MiNEN) | 1.8 | 0.5-6.47 | | 0.369 |
| Hepatic Metastases (Yes vs No) | 2.6 | 0.32-20.98 | | 0.371 |
| Lung Metastases (Yes vs No) | 0.84 | 0.18-3.94 | | 0.826 |
| Bone Metastases (Yes vs No) | 0.45 | 0.14-1.44 | | 0.177 |
| Elevated NSE (Yes vs No) | 0.93 | 0.33-2.64 | | 0.894 |
| Elevated CA199 (Yes vs No) | 0.98 | 0.21-4.54 | | 0.979 |
| Elevated CEA (Yes vs No) | 0.79 | 0.17-3.62 | | 0.764 |
| Ki67 | 0.21 | 0-13.85 | | 0.464 |
| Ki67 (≥55% vs <55%) | 1.83 | 0.4-8.35 | | 0.433 |
| PDL1(CPS) (≥1 vs <1) | 0.76 | 0.09-6.17 | | 0.794 |
| TMB (Low vs High) | 0.75 | 0.25-2.19 | | 0.595 |
| Surgery (Yes vs No) | 1.69 | 0.61-4.67 | | 0.313 |
| First-line (EP vs Non-EP) | 4.17 | 1.28-13.64 | | 0.018 |
| Multivariate analysis | | | | |
| Site (Rectum vs Colon) | 0.69 | 0.23-2.05 | | 0.502 |
| Pathology (SCNEC vs Non-SCNEC) | 1.50 | 0.50-4.48 | | 0.465 |
| First-line (EP vs Non-EP) | 3.88 | 1.16-13.00 | | 0.028 |

# TableS8. Univariate/Multivariate Cox proportional hazard regression analysis of second-line therapy of Type II GEPNEC.

| Variable | Factors | Hazard Ratio | CI95^*^ | P Value |
| --- | --- | --- | --- | --- |
| Univariate analysis | | | | |
| Primary site | Rectum vs Colon | 0.7 | 0.24-2.04 | 0.516 |
| Gender | Male vs Female | 1.56 | 0.33-7.31 | 0.569 |
| Age |  | 1 | 0.94-1.07 | 0.884 |
| Pathology | SCNEC vs Non-SCNEC | 1.07 | 0.33-3.49 | 0.91 |
| Diagnosis | NEC vs MiNEN | 1.25 | 0.38-4.1 | 0.714 |
| Hepatic Metastases | Yes vs No | 1.57 | 0.2-12.41 | 0.669 |
| Lung Metastases | Yes vs No | 0.75 | 0.16-3.46 | 0.711 |
| Bone Metastases | Yes vs No | 0.2 | 0.04-0.97 | 0.045 |
| NSE | Elevated vs No | 0.69 | 0.21-2.25 | 0.543 |
| CA199 | Elevated vs No | 4.58 | 0.63-33.03 | 0.131 |
| CEA | Elevated vs No | 2.3 | 0.71-7.4 | 0.163 |
| Ki67 |  | 0.11 | 0-9.09 | 0.33 |
| Second-line | Immunotherapy/Target vs Chemotherapy | 0.28 | 0.08-0.98 | 0.047 |
| TMB | Low vs High | 2.09 | 0.56-7.81 | 0.272 |
| Surgery history | Yes vs No | 1.49 | 0.41-5.43 | 0.547 |
| Multivariate analysis | | | | |
| Primary site | Rectum vs Colon | 2.90 | 0.48-7.54 | 0.357 |
| Pathology | SCNEC vs Non-SCNEC | 0.46 | 0.14-1.49 | 0.197 |
| Bone Metastases | Yes vs No | 0.27 | 0.04-1.94 | 0.194 |
| Second-line | Immunotherapy/Target vs Chemotherapy | 0.47 | 0.08-2.77 | 0.406 |

^*:^ Confidential interval.

# TableS9. Targetable gene alteration events and their indicated therapy across different GEPNEC

|  |  | F-NEC | MH-NEC | Other | FDA/NMPA granted drugs |
| --- | --- | --- | --- | --- | --- |
| ALK | 1 | 0 | 1 | 0 | Crizotinib, Lorlatinib, Ceritinib, Alectinib, Brigatinib |
| BRAF p.V600E | 2 | 1 | 1 | 0 | Vemurafenib,Dabrafenib,Encorafenib |
| BRCA1/2 | 12 | 8 | 4 | 0 | Olaparib, Rucaparib, Niraparib,Talazoparib |
| ERBB2 | 1 | 1 | 1 | 0 | Trastuzumab, Pertuzumab, T-DM1,Trastuzumab Deruxtecan(DS-8201),Neratinib,Pyrotinib,Lapatinib,Tucatinib |
| KRAS p.G12C | 1 | 1 | 0 | 0 | Sotorasib, Adagrasib, JNJ-74699157, |
| MET | 4 | 3 | 0 | 1 | Amivantamab,Tepotinib,Capmatinib |
| PIK3CA p.E454K | 1 | 0 | 0 | 1 | Alpelisib, Capivasertib, Ribociclib, Everolimus, Pictilisib, Fulvestrant |
| RET mutation | 3 | 1 | 0 | 2 | Preseletinib, Selpercatinib, Pralsetinib, Cabozantinib, Vandetanib, |
| Microsatellite instability | 4 | 0 | 2 | 1 | Pembrolizumab, Nivolumab, Ipilimumab,Sintilimab,Tislelizumab,Toriplimab,Camrelizumab,Envafolomab |
| TMB-H | 21 | 12 | 5 | 4 | Pembrolizumab, Nivolumab, Ipilimumab, Sintilimab,Tislelizumab,Toriplimab,Camrelizumab,Envafolomab |

# TableS10. Univariate/multivariate Cox regression analysis of PFS for targetable patients with target therapy.

| **Variable** | **Factors** | **Hazard Ratio** | **CI95^*^** | **P Value** |
| --- | --- | --- | --- | --- |
| **Univariate analysis** | | | | |
| Pathology | SCNEC vs Non-SCNEC | 0.9 | 0.5-1.6 | 0.714 |
| Site | Colon vs Bile tract | 1.29 | 0.36-4.64 | 0.692 |
|  | Duodenum vs Bile tract | 0.9 | 0.2-4.07 | 0.896 |
|  | Esophagus vs Bile tract | 1.98 | 0.49-8.05 | 0.34 |
|  | Stomach vs Bile tract | 0.94 | 0.32-2.77 | 0.905 |
|  | Other vs Bile tract | 0.27 | 0.06-1.27 | 0.099 |
|  | Pancreas vs Bile tract | 1.4 | 0.37-5.35 | 0.622 |
|  | Rectum vs Bile tract | 0.91 | 0.23-3.51 | 0.888 |
| Age | | 0.99 | 0.97-1.02 | 0.655 |
| Origin | MH-NEC vs F-NEC | 1.05 | 0.52-2.09 | 0.9 |
|  | Other-NEC vs F-NEC | 0.27 | 0.08-0.91 | 0.034 |
| Gender | Male vs Female | 1.58 | 0.86-2.87 | 0.138 |
| Diagnosis | NEC vs MiNEN | 0.93 | 0.47-1.88 | 0.849 |
| Ki67 | | 2.64 | 0.49-14.05 | 0.256 |
| Hepatic Metastases | Yes vs No | 1.64 | 0.85-3.17 | 0.14 |
| Lung Metastases | Yes vs No | 0.8 | 0.36-1.8 | 0.598 |
| Bone Metastases | Yes vs No | 0.52 | 0.21-1.24 | 0.138 |
| Elevated NSE | Yes vs No | 0.92 | 0.52-1.62 | 0.769 |
| Elevated CA199 | Yes vs No | 1.27 | 0.64-2.49 | 0.496 |
| Elevated CEA | Yes vs No | 0.79 | 0.36-1.72 | 0.555 |
| TMB | Low vs High | 1.45 | 0.77-2.73 | 0.252 |
| Surgery history | Yes vs No | 1.19 | 0.65-2.16 | 0.578 |
| PDL1(CPS) | >=1 vs <1 | 0.52 | 0.27-0.98 | 0.043 |
| Target therapy | Yes vs No | 0.4 | 0.18-0.89 | 0.024 |
| Multivariate analysis | | | | |
| Target therapy | Yes vs No | 0.44 | 0.20-0.97 | 0.042 |
| PDL1(CPS) | >=1 vs <1 | 0.57 | 0.30-1.08 | 0.083 |

# TableS11. The association of genotypes and efficacy to different first-line/second-line regimens.

|  |  | ORR | | PFS (months) | |
| --- | --- | --- | --- | --- | --- |
| First-line | | | | | |
|  |  | CT^a^ | EP | CT | EP |
| MH-NEC | Type II | 2/8,25% | 1/12,8.3% | 7.7(4.1,NA) | 3(2.7,NA) |
|  | non-Type II | 4/18,22% | 6/14,42.9% | 4.9(3.1,7.9) | 4.5(2.3,NA) |
| F-NEC | Type III | 4/12,33% | 15/44,34% | 6.0(3.1,NA) | 5.3(4.4,6.8) |
|  | non-Type III | 13/28,46% | 17/52,33% | 5.0(4.0,10.0) | 4.8(3.9,6.0) |
| Second-line | | | | | |
|  |  | CT^b^ | Immunotherapy | CT | Immunotherapy |
| MH-NEC | Type II | 1/10,10% | 2/5,40% | 2.5(1.5,NA) | 6.1(3.0,NA) |
|  | non-Type II | 4/13,31% | 1/7,14% | 3.4(1.8,NA) | 1,9(1.3,NA) |
| F-NEC | Type III | 8/36,22% | 6/19,32% | 3.5(2.5,5.7) | 3.0(2.4,NA) |
|  | non-Type III | 6/28,21% | 2/13,15% | 2.2(1.8,4.4) | 2.0(1.7,NA) |

**Abbreviations:** MH-NEC: midgut/hindgut NEC; F-NEC: foregut NEC. CT: chemotherapy.

**Annotations:** ^a^: non-EP chemotherapy ^b:^ All chemotherapy ^c^: Immunotherapy referred to those with immunotherapy, anti-angiogenesis therapy or its combinations.

# TableS12. List of genes in the 425 panel.

| ABCB1 (MDR1) | ABCC2 (MRP2) | ADGRB3 (BAI3) | ADH1B | AFDN (MLLT4) | AIP | AKT1 | AKT2 | AKT3 | ALDH2 |
| --- | --- | --- | --- | --- | --- | --- | --- | --- | --- |
| ALK | AMER1 | APC | AR | ARAF | ARID1A | ARID1B | ARID2 | ARID5B | ASCL4 |
| ASXL1 | ATF1 | ATIC | ATM | ATR | ATRX | AURKA | AURKB | AXIN2 | AXL |
| B2M | BAD | BAK1 | BAP1 | BARD1 | BAX | BCL2 | BCL2L11 (BIM) | BCR | BIRC3 |
| BLM | BMPR1AA62 | BRAF | BRCA1 | BRCA2 | BRD4 | BRIP1 | BTG2 | BTK | BUB1B |
| CASP8 | CBL | CBLB | CCN6 (WISP3) | CCND1 | CCNE1 | CD274 (PD-L1) | CD74 | CDA | CDC73 |
| CDH1 | CDK10 | CDK12 | CDK4 | CDK6 | CDK8 | CDKN1A | CDKN1B | CDKN1C | CDKN2A |
| CDKN2B | CDKN2C | CEBPA | CEP57 | CHD4 | CHD8 | CHEK1 | CHEK2 | CREBBP | CRKL |
| CSF1R | CTCF | CTLA4 | CTNNB1 | CUL3 | CUX1 | CXCR4 | CYLD | CYP19A1 | CYP2A13 |
| CYP2A6 | CYP2A7 | CYP2B6*6 | CYP2C19*2 | CYP2C9*3 | CYP2D6 | CYP3A4*4 | CYP3A5 | CYSLTR2 | DAXX |
| DDR2 | DENND1A | DHFR | DICER1 | DLL3 | DNMT3A | DOT1L | DPYD | DTL (CDT2) | DUSP2 |
| EGFR | EIF1AX | EMSY (c11orf30) | EP300 | EPAS1 | EPCAM | EPHA2 | EPHA3 | EPHA5 | ERBB2 (HER2) |
| ERBB3 | ERBB4 | ERBIN (ERBB2IP) | ERCC1 | ERCC2 | ERCC3 | ERCC4 | ERCC5 | ESR1 | ETV1 |
| ETV4 | ETV5 | ETV6 | EWSR1 | EXT1 | EXT2 | EZH2 | EZR | FANCA | FANCC |
| FANCD2 | FANCE | FANCF | FANCG | FANCI | FANCL | FANCM | FAT1 | FBXW7 | FGF19 |
| FGFR1 | FGFR2 | FGFR3 | FGFR4 | FH | FLCN | FLT1 (VEGFR1) | FLT3 | FLT4 | FOXA1 |
| FOXL2 | FOXP1 | FRG1 | GATA1 | GATA2 | GATA3 | GATA4 | GATA6 | GNA11 | GNAQ |
| GNAS | GRIN2A | GRM3 | GRM8 | GSTM1 | GSTM4 | GSTP1 | GSTT1 | HDAC2 | HDAC9 |
| HGF | HLA-A | HNF1A | HNF1B | HRAS | IDH1 | IDH2 | IFNA6 | IFNB1 | IFNE |
| IFNG | IFNGR1 | IFNGR2 | IGF1R | IGF2 | IKBKE | IKZF1 | IL7R | INPP4B | IRF2 |
| JAK1 | JAK2 | JAK3 | JARID2 | JUN | KDM5A | KDR (VEGFR2) | KEAP1 | KIF1B | KIT |
| KITLG | KLLN | KMT2A (MLL) | KMT2B | KMT2C | KMT2D (MLL2) | KRAS | LHCGR | LMO1 | LRP1B |
| LYN | LZTR1 | MAP2K1 (MEK1) | MAP2K2 (MEK2) | MAP2K4 | MAP3K1 | MAP3K4 | MAX | MCL1 | MDM2 |
| MDM4 | MECOM | MED12 | MEF2B | MEN1 | MET | MGMT | MITF | MLH1 | MLH3 |
| MLLT1 | MLLT3 | MPL | MRE11 (MRE11A) | MSH2 | MSH6 | MTHFR | MTOR | MUTYH | MYC |
| MYCL (MYCL1) | MYCN | MYD88 | MYH9 | NAT1 | NBN | NCOR1 | NF1 | NF2 | NFE2L2 |
| NFKBIA | NKX2-1 | NOTCH1 | NOTCH2 | NOTCH3 | NPM1 | NQO1 | NRAS | NRG1 | NSD1 |
| NTRK1 | NTRK2 | NTRK3 | NUTM1 | PAK3 | PALB2 | PALLD | PARP1 | PARP2 | PAX5 |
| PBRM1 | PDCD1 (PD1) | PDCD1LG2 (PD-L2) | PDE11A | PDGFRA | PDGFRB | PDK1 | PGR | PHOX2B | PIK3C3 |
| PIK3CA | PIK3CD | PIK3R1 | PIK3R2 | PKHD1 | PLAG1 | PLCB4 | PLK1 | PMS1 | PMS2 |
| POLD1 | POLD3 | POLE | POLH | POT1 | PPARD | PPP2R1A | PRDM1 | PREX2 | PRF1 |
| PRKACA | PRKAR1A | PRKCI | PRKDC | PRKN (PARK2) | PRSS1 | PRSS3 | PTCH1 | PTEN | PTK2 |
| PTPN11 | PTPN13 | QKI | RAC1 | RAC3 | RAD50 | RAD51 | RAD51B | RAD51C | RAD51D |
| RAD54L | RAF1 | RARA | RARG | RASGEF1A | RB1 | RECQL4 | RELN | RET | RHOA |
| RICTOR | RNF43 | ROS1 | RPTOR | RRM1 | RUNX1 | RUNX1T1 | SBDS | SDC4 | SDHA |
| SDHB | SDHC | SDHD | SEPTIN9 (SEPT9) | SETBP1 | SETD2 | SF3B1 | SGK1 | SKP2 | SLC34A2 |
| SLC3A2 | SMAD2 | SMAD3 | SMAD4 | SMAD7 | SMARCA4 | SMARCB1 | SMO | SOCS1 | SOS1 |
| SOX2 | SPOP | SPRED1 | SPRY4 | SRC | SRSF2 | SRY | STAG2 | STAT3 | STK11 |
| STMN1 | SUFU | TACC3 | TAP1 | TAP2 | TEK | TEKT4 | TERC | TERT | TET2 |
| TGFBR2 | THADA | TMEM127 | TMPRSS2 | TNFAIP3 | TNFRSF11A | TNFRSF14 | TNFRSF19 | TNFSF11 | TOP1 |
| TOP2A | TP53 | TP63 | TPMT | TSC1 | TSC2 | TSHR | TTF1 | TUBB3 | TYMS |
| U2AF1 | UGT1A1 | VAMP2 | VEGFA | VHL | WAS | WRN | WT1 | XPA | XPC |
| XRCC1 | XRCC2 | YAP1 | ZNF217 | ZNF703 |  |  |  |  |  |

# Reference

1. Venizelos, A., et al., *The molecular characteristics of high-grade gastroenteropancreatic neuroendocrine neoplasms.* Endocr Relat Cancer, 2021.

2. Puccini, A., et al., *Comprehensive Genomic Profiling of Gastroenteropancreatic Neuroendocrine Neoplasms (GEP-NENs).* Clinical Cancer Research, 2020. **26**(22): p. 5943-5951.

3. Sahnane, N., et al., *Microsatellite unstable gastrointestinal neuroendocrine carcinomas: A new clinicopathologic entity.* Endocrine-Related Cancer, 2015. **22**(1): p. 35-45.

4. Busico, A., et al., *Gastroenteropancreatic High-Grade Neuroendocrine Neoplasms: Histology and Molecular Analysis, Two Sides of the Same Coin.* Neuroendocrinology, 2020. **110**(7-8): p. 616-629.

5. Gerard, L., et al., *ctDNA in neuroendocrine carcinoma of gastroenteropancreatic origin or of unknown primary: the CIRCAN-NEC pilot study.* Neuroendocrinology, 2020.

6. Vijayvergia, N., et al., *Molecular profiling of neuroendocrine malignancies to identify prognostic and therapeutic markers: A Fox Chase Cancer Center Pilot Study.* British Journal of Cancer, 2016. **115**(5): p. 564-570.

7. Olevian, D.C., et al., *Colorectal poorly differentiated neuroendocrine carcinomas frequently exhibit BRAF mutations and are associated with poor overall survival.* Human Pathology, 2016. **49**: p. 124-134.

8. Takizawa, N., et al., *Molecular characteristics of colorectal neuroendocrine carcinoma; Similarities with adenocarcinoma rather than neuroendocrine tumor.* Human Pathology, 2015. **46**(12): p. 1890-1900.

9. Lee, S.M. and C.O. Sung, *Comprehensive analysis of mutational and clinicopathologic characteristics of poorly differentiated colorectal neuroendocrine carcinomas.* Scientific reports, 2021. **11**(1): p. 6203-6203.

10. Capdevila, J., et al., *Epigenetic EGFR gene repression confers sensitivity to therapeutic BRAFV600E blockade in colon neuroendocrine carcinomas.* Clinical Cancer Research, 2020. **26**(4): p. 902-909.

11. Shamir, E.R., et al., *Identification of high-risk human papillomavirus and Rb/E2F pathway genomic alterations in mutually exclusive subsets of colorectal neuroendocrine carcinoma.* Modern Pathology, 2019. **32**(2): p. 290-305.

12. Woischke, C., et al., *In-depth mutational analyses of colorectal neuroendocrine carcinomas with adenoma or adenocarcinoma components.* Modern Pathology, 2017. **30**(1): p. 95-103.

13. Konukiewitz, B., et al., *Pancreatic neuroendocrine carcinomas reveal a closer relationship to ductal adenocarcinomas than to neuroendocrine tumors G3.* Human Pathology, 2018. **77**: p. 70-79.

14. Liu, F., et al., *Whole-exome mutational landscape of neuroendocrine carcinomas of the gallbladder.* Signal Transduction and Targeted Therapy, 2021. **6**(1).

15. Li, R., et al., *Multi-omics profiling of primary small cell carcinoma of the esophagus reveals RB1 disruption and additional molecular subtypes.* Nat Commun, 2021. **12**(1): p. 3785.

16. Meder, L., et al., *NOTCH, ASCL1, p53 and RB alterations define an alternative pathway driving neuroendocrine and small cell lung carcinomas.* International Journal of Cancer, 2016. **138**(4): p. 927-938.

17. Bergsland, E.K., et al., *Genomic profiling to distinguish poorly differentiated neuroendocrine carcinomas arising in different sites.* J Clin Oncol, 2016. **34**(15).

18. Makuuchi, R., et al., *Comprehensive analysis of gene mutation and expression profiles in neuroendocrine carcinomas of the stomach.* Biomedical Research-Tokyo, 2017. **38**(1): p. 19-27.

19. Scardoni, M., et al., *Mixed adenoneuroendocrine carcinomas of the gastrointestinal tract: Targeted next-generation sequencing suggests a monoclonal Origin of the Two Components.* Neuroendocrinology, 2014. **100**(4): p. 310-316.

20. Koh, J., et al., *Comprehensive genetic features of gastric mixed adenoneuroendocrine carcinomas and pure neuroendocrine carcinomas.* Journal of Pathology, 2021. **253**(1): p. 94-105.
