# Supplementary figures and images for: Prognostic and therapeutic roles of specific genotypes through target-gene sequencing on gastroenteropancreatic neuroendocrine carcinoma
Source: Oncologist. 2026 May 8;31(6):oyag185. doi: 10.1093/oncolo/oyag185 (PMC13215382; doi:10.1093/oncolo/oyag185)

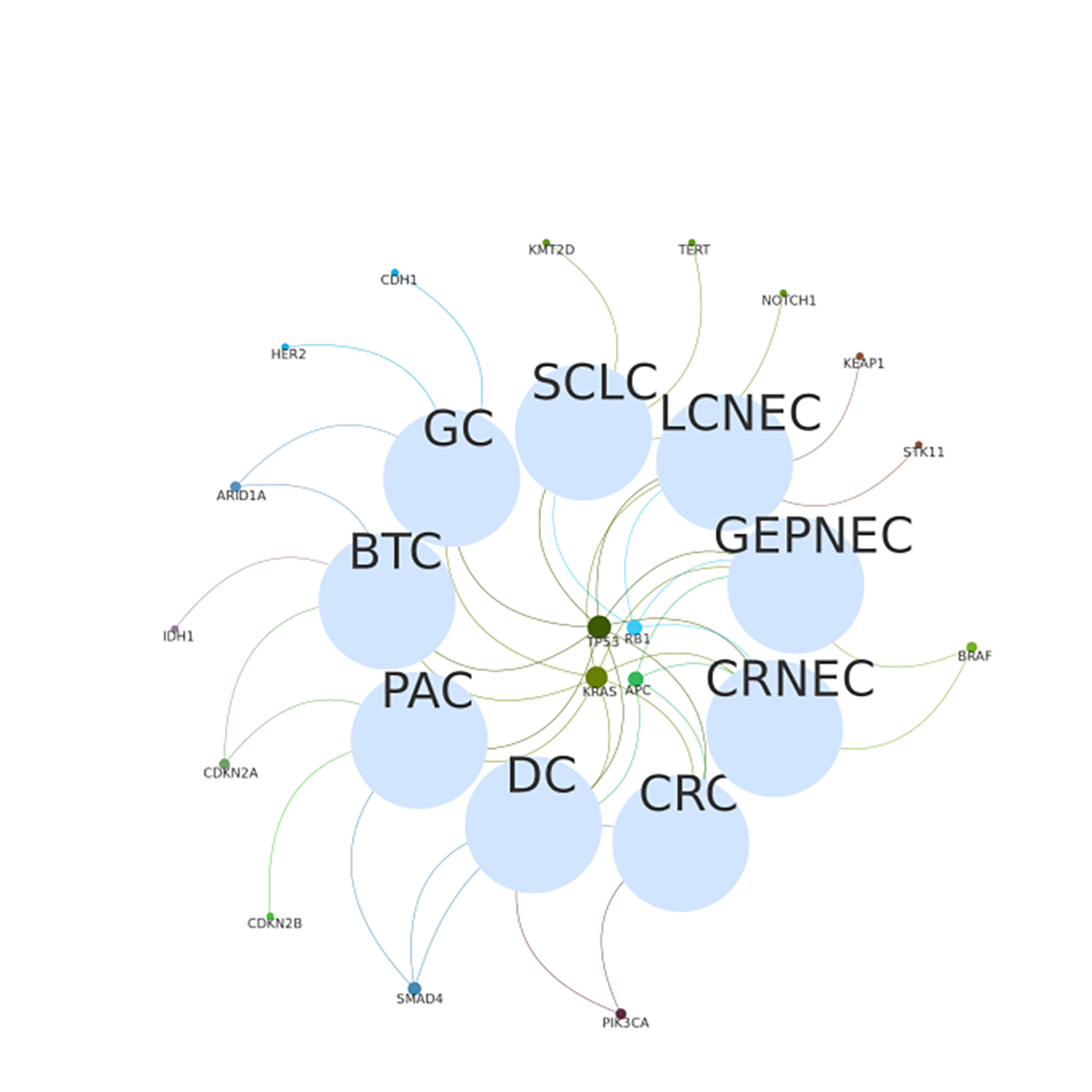

Supplement: oyag185_Supplementary_Data [file oyag185_supplementary_data.zip › FigureS4 .tif]

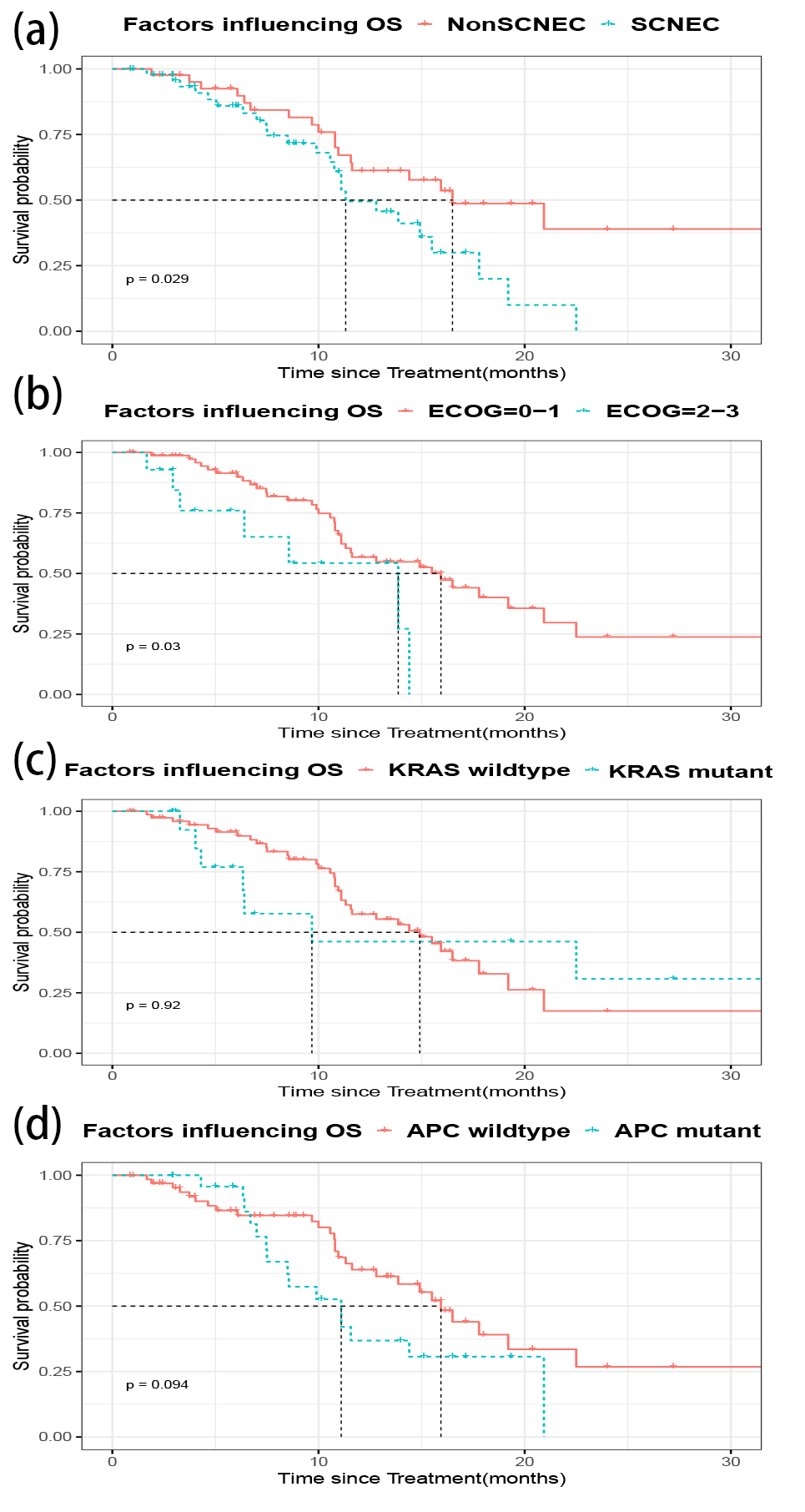

Supplement: oyag185_Supplementary_Data [file oyag185_supplementary_data.zip › FigureS5.tif]

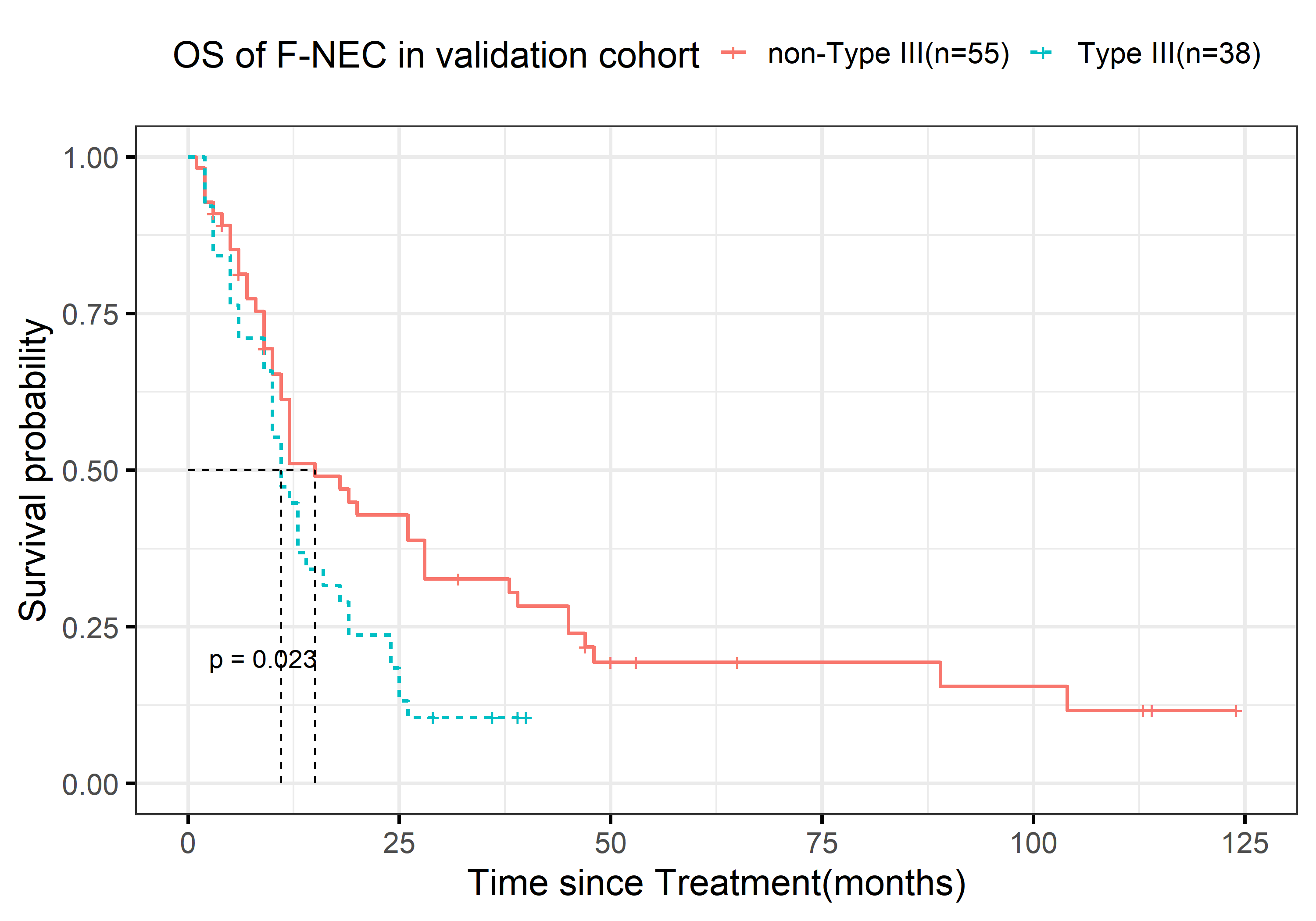

Supplement: oyag185_Supplementary_Data [file oyag185_supplementary_data.zip › FigureS6.bmp]

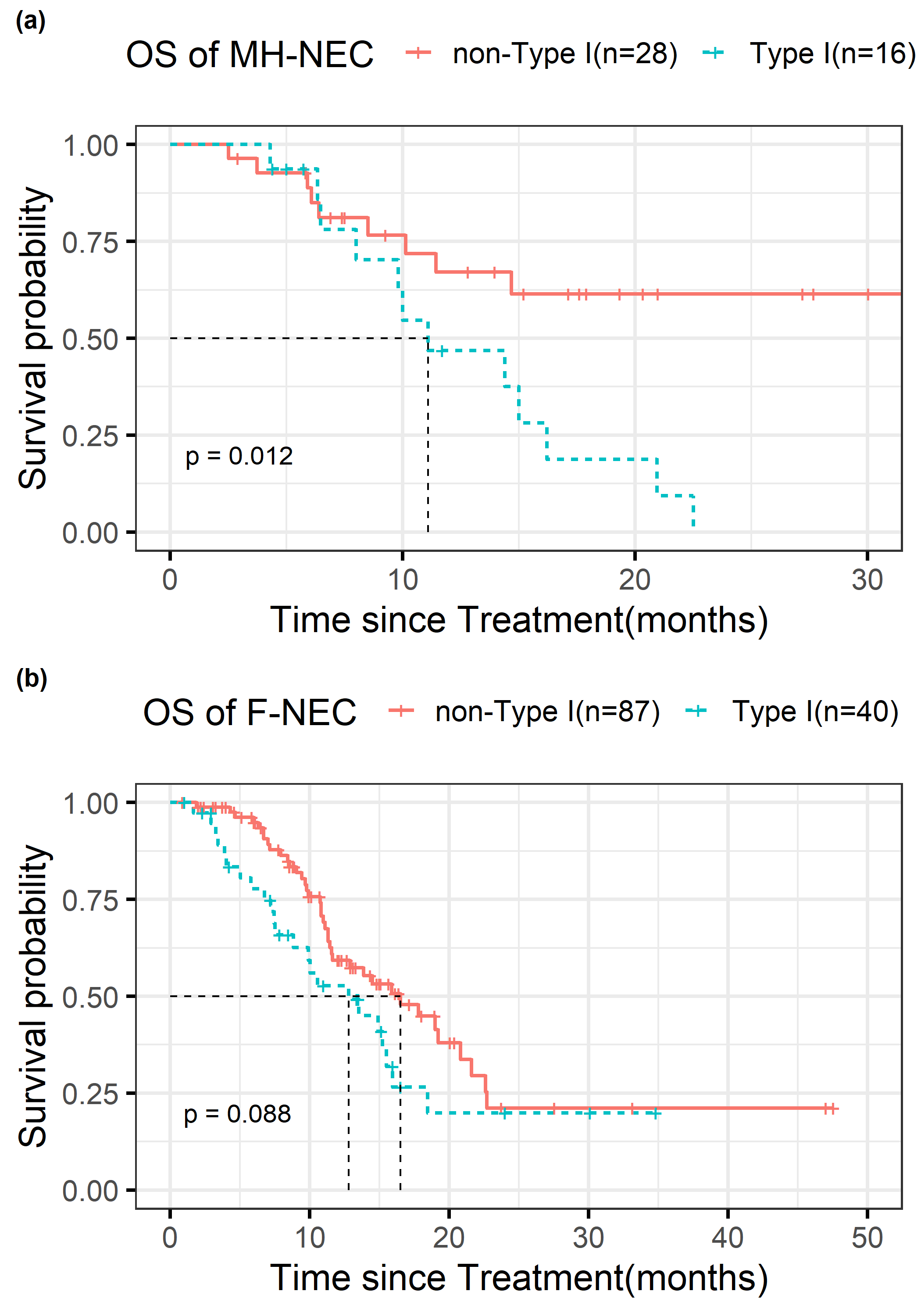

Supplement: oyag185_Supplementary_Data [file oyag185_supplementary_data.zip › FigureS7.bmp]

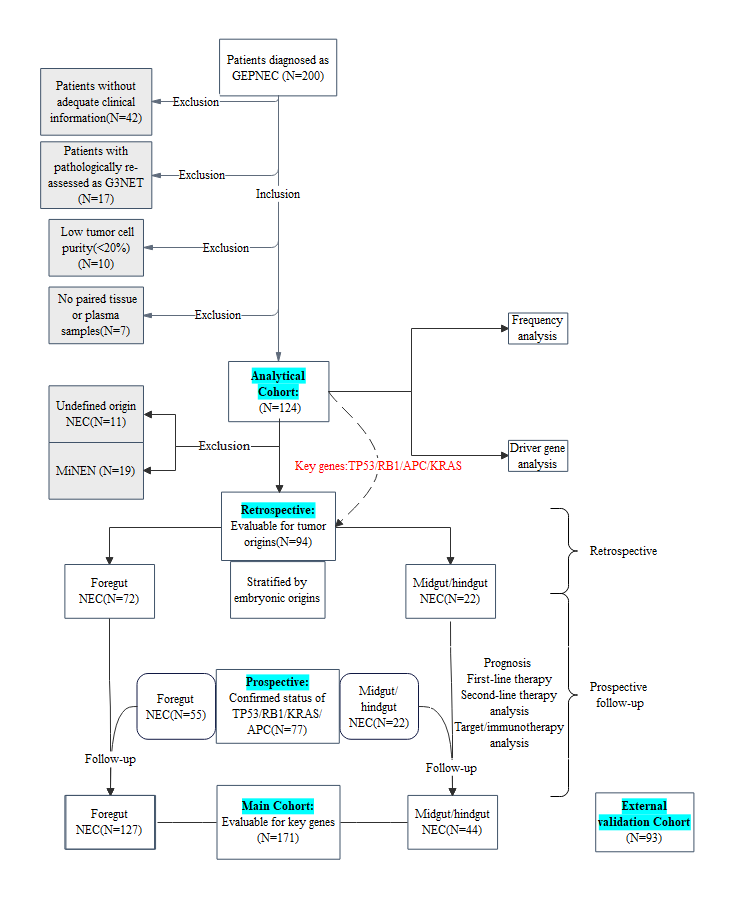

Supplement: oyag185_Supplementary_Data [file oyag185_supplementary_data.zip › FigureS1.tiff]

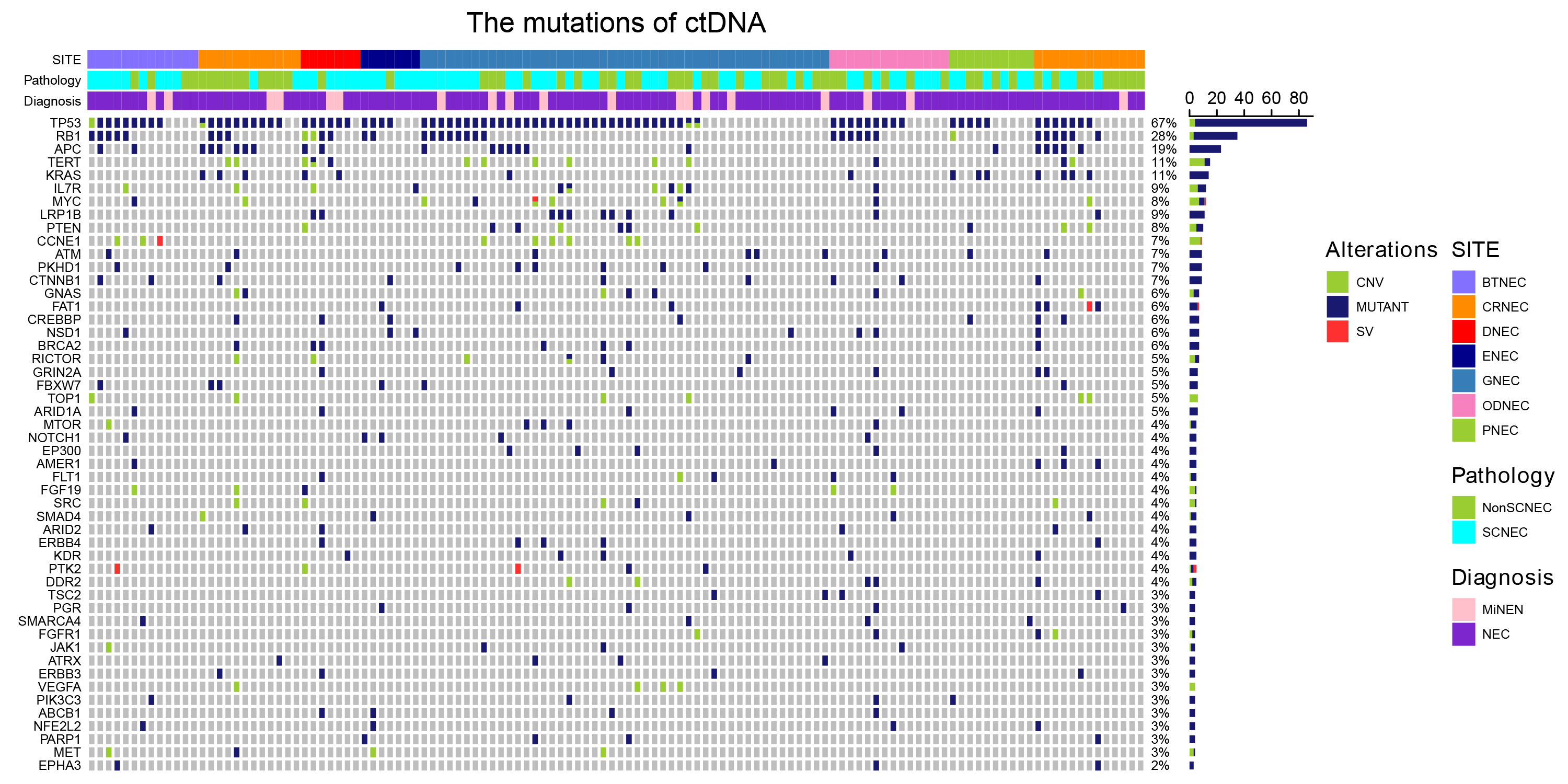

Supplement: oyag185_Supplementary_Data [file oyag185_supplementary_data.zip › FigureS2.tif]
